# Collider Interplay for Supersymmetry, Higgs and Dark Matter

**DOI:** 10.1140/epjc/s10052-015-3675-3

**Published:** 2015-10-01

**Authors:** O. Buchmueller, M. Citron, J. Ellis, S. Guha, J. Marrouche, K. A. Olive, K. de Vries, Jiaming Zheng

**Affiliations:** High Energy Physics Group, Blackett Lab., Imperial College, Prince Consort Road, London, SW7 2AZ UK; Theoretical Particle Physics and Cosmology Group, Department of Physics, King’s College London, London, WC2R 2LS UK; Physics Department, CERN, 1211 Geneva 23, Switzerland; BITS Pilani, Goa Campus, Goa, India; William I. Fine Theoretical Physics Institute, School of Physics and Astronomy, Univ. of Minnesota, Minneapolis, MN 55455 USA

## Abstract

We discuss the potential impacts on the CMSSM of future LHC runs and possible $e^+ e^-$ and higher-energy proton–proton colliders, considering searches for supersymmetry via  $/ E_T$ events, precision electroweak physics, Higgs measurements and dark matter searches. We validate and present estimates of the physics reach for exclusion or discovery of supersymmetry via $/ E_T$ searches at the LHC, which should cover the low-mass regions of the CMSSM parameter space favoured in a recent global analysis. As we illustrate with a low-mass benchmark point, a discovery would make possible accurate LHC measurements of sparticle masses using the MT2 variable, which could be combined with cross-section and other measurements to constrain the gluino, squark and stop masses and hence the soft supersymmetry-breaking parameters $m_0, m_{1/2}$ and $A_0$ of the CMSSM. Slepton measurements at CLIC would enable $m_0$ and $m_{1/2}$ to be determined with high precision. If supersymmetry is indeed discovered in the low-mass region, precision electroweak and Higgs measurements with a future circular $e^+ e^-$ collider (FCC-ee, also known as TLEP) combined with LHC measurements would provide tests of the CMSSM at the loop level. If supersymmetry is not discovered at the LHC, it is likely to lie somewhere along a focus-point, stop-coannihilation strip or direct-channel *A* / *H* resonance funnel. We discuss the prospects for discovering supersymmetry along these strips at a future circular proton–proton collider such as FCC-hh. Illustrative benchmark points on these strips indicate that also in this case FCC-ee could provide tests of the CMSSM at the loop level.

## Introduction

The first run of the LHC at 7 and 8 TeV has framed the agenda for its future runs, and for possible future colliders. The CMS and ATLAS experiments have discovered a Higgs boson [1, 2], but have found no sign of supersymmetry or any other physics beyond the Standard Model [3, 4]. Present and future studies of the Higgs boson can be used to constrain scenarios for new physics, as can other high-precision low-energy measurements and cosmological constraints. We address in this paper the prospects for discovering supersymmetry during future runs of the LHC at 13/14 TeV in light of the indirect information currently provided by the Higgs and other measurements, and consider possible scenarios for discovering or measuring supersymmetry at proposed future linear and circular colliders, either directly or indirectly, showing how the various colliders may complement each other.

Our study is within the minimal supersymmetric extension of the Standard Model with soft supersymmetry-breaking parameters constrained to be universal at a high input scale, the CMSSM [5–19]. This model is not imposed by top-down considerations based on string, M- or F-theory, nor is it required by bottom-up considerations such as the limits on flavour-changing neutral interactions. However, it is the simplest supersymmetric model, so its phenomenology is relatively unambiguous. As such, it provides a convenient benchmark for considering the interplay between different high-energy colliders.

One of the most important constraints that we take into account is the bound on the density of cold dark matter, which provides interesting constraints on the parameters of the CMSSM. In particular, requiring that the relic density of the lightest supersymmetric particle (LSP), assumed here to be the lightest neutralino $\chi$ [20, 21], falls within the range allowed by astrophysics and cosmology can be used to provide important constraints, including upper limits, on the soft supersymmetry-breaking mass parameters in the CMSSM, and hence sparticle masses [22–30].

The LHC measurement of the Higgs mass already provides a significant constraint on the parameter space of the CMSSM, favouring sparticle masses that are consistent with the non-observation of supersymmetric particles at the LHC in Run 1 [29–58]. The starting point for our analysis is a recent global fit to the CMSSM model parameters [33], using these measurements as well as precision electroweak and flavour observables, as well as direct constraints on the interactions of the LSP with ordinary matter.

In order to evaluate the potential of future LHC runs to probe the CMSSM, we extrapolate the sensitivities of gluino, squark and stop searches at LHC Run 1 at 7 and 8 TeV to estimate LHC capabilities with 300 or 3000/fb of data at 13/14 TeV. We find that such data sets should permit the LHC experiments to discover supersymmetry if it has CMSSM parameters within the low-mass region favoured by the global fit [33]. Assuming optimistically that they are given by the best current fit in this low-mass region, we then discuss how accurately the LHC experiments could measure the gluino, squark and stop masses, and hence the CMSSM soft supersymmetry-breaking parameters $m_0, m_{1/2}$ and $A_0$.

In this optimistic scenario where Nature is described by the CMSSM in the low-mass region, experiments at the proposed CLIC $e^+ e^-$ collider at 3 TeV in the centre of mass [59, 60] would be able to produce and measure very accurately the masses and other properties of the sleptons and the lighter gauginos, enabling, for example, high-precision determinations of the soft supersymmetry-breaking parameters $m_0$ and $m_{1/2}$ of the CMSSM. An $e^+ e^-$ collider with an energy 1 TeV could also explore parts of the low-mass region, e.g., pair-producing the lighter stau slepton at the low-mass best-fit point. On the other hand, $e^+ e^-$ colliders with energies below 500 GeV in the centre of mass would not be able to produce and measure sparticles directly.

As we discuss, measurements of *Z*-boson [61] and Higgs couplings [62–64] do not as yet provide strong supplementary constraints on supersymmetric models such as the CMSSM. However, future higher-precision measurements could be used to constrain the CMSSM parameters indirectly. In particular, if Nature is indeed described by the CMSSM with parameters in the low-mass region, measurements of the *Z* and Higgs boson at the proposed high-luminosity circular $e^+ e^-$ collider FCC-ee (TLEP) [65] could be used, in conjunction with the LHC measurements, to test this supersymmetric model at the quantum level, as we illustrate in the specific example of the best-fit low-mass point from [33]. As an aside, we also show how, again in the optimistic low-mass scenario, high-precision *Z* measurements at FCC-ee (TLEP) could be used to probe models of supersymmetric grand unification.

On the other hand, in the pessimistic scenario where the LHC does not discover supersymmetry but only establishes 95 % CL lower limits on particle masses, we consider the prospects for discovering supersymmetry directly at a future higher-energy circular proton–proton collider such as FCC-hh [66]. For some studies with similar motivations, see [67–71], for some other studies of supersymmetry at a 100-TeV *pp* collider, see [72–76] or finding indirect evidence for supersymmetry via high-precision $e^+ e^-$ measurements. Within the CMSSM, high-scale supersymmetric models can be found along narrow strips where stop-neutralino coannihilation is important [58], or in the focus-point region [56, 57], and we analyse the prospects of direct and indirect measurements along these strips. Studies of illustrative benchmark points along these strips indicate that the combination of direct FCC-hh and indirect FCC-ee measurements could test supersymmetry at the loop level also in this pessimistic case.

The layout of this paper is as follows. In Sect. 2 we discuss the extrapolations of current LHC sparticle search sensitivities to future LHC runs. Then, in Sect. 3, we discuss possible LHC measurements of particle masses in the optimistic low-mass best-fit scenario. Section 4 contains our discussion of $e^+ e^-$ probes of supersymmetry in this optimistic scenarios, including direct searches at CLIC as well as indirect constraints due to high-precision *Z* and Higgs measurements at FCC-ee (TLEP). The pessimistic high-mass scenarios in which the LHC does not discover supersymmetry are discussed in Sect. 5, where we consider the prospects for direct discovery with FCC-hh as well as indirect measurements with FCC-ee (TLEP). Finally, our conclusions are summarised in Sect. 6.

## Extrapolations of current LHC sparticle search sensitivities to higher energy and luminosity

**Fig. 1:**
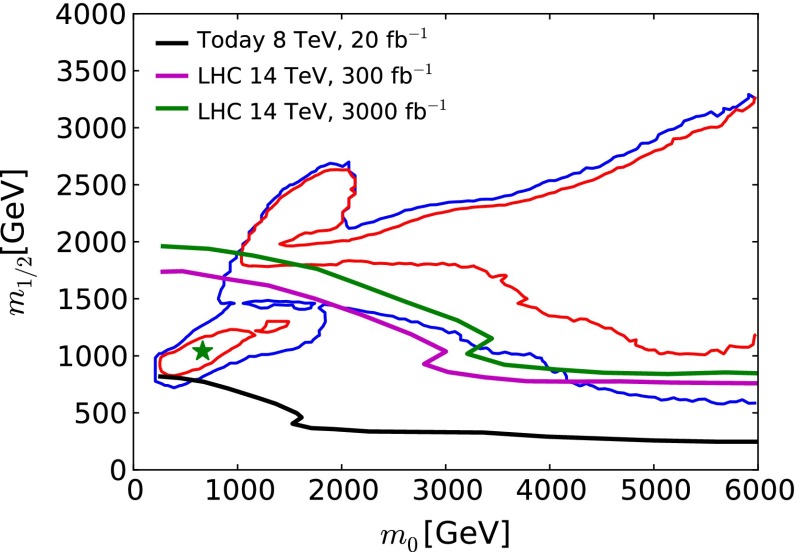
The $(m_0, m_{1/2})$ plane in the CMSSM. The $\Delta \chi ^2 =$ 2.30 (68 % CL) and 5.99 (95 % CL) regions found in recent global fits are bounded by *solid red* and *blue lines*, respectively. The best-fit point in the low-mass ‘Crimea’ regions is indicated by a *filled green star*. Also shown as *solid black* (*purple*, *green*) *lines* are the sensitivities of LHC  $/ E_T$ searches for exclusions at the 95 % CLs with 20/fb of data at 8 TeV (300, 3000/fb of data at 14 TeV). The *purple contour* is expected to coincide (within uncertainties) with the 5-$\sigma$ discovery contour at the LHC with 3000/fb of data at 14 TeV

The baseline for our studies is provided by a recent global fit to the parameters of the CMSSM [33].^1^ In addition to the ATLAS search for jets + $/ E_T$ events with $\sim$20/fb of 8 TeV data [3, 4], these global fits included the measurement of $m_h$ [1, 2, 85] (which was related to the CMSSM parameters via calculations using FeynHiggs 2.10.0 [86–91]), electroweak precision observables and $g_\mu - 2$ [92, 93], precision flavour observables including $b \rightarrow s \gamma$ [94–97] and $B_{s,d} \rightarrow \mu ^+ \mu ^-$ [98–103], and dark matter observables including the direct LUX constraint on dark matter scattering [104] and the total cold dark matter density [105]. These measurements were combined into a global $\chi ^2$ likelihood function, whose projection on the $(m_0, m_{1/2})$ plane of the CMSSM is displayed in Fig. 1. In this and subsequent figures, we marginalise over the other CMSSM parameters $\tan \beta$ and $A_0$. We display in red and blue, respectively, $\Delta \chi ^2 = 2.30$ and 5.99 contours (which we use as proxies for 68 and 95 % CL contours). For each set of $(m_0, m_{1/2})$ values within these contours, there is some choice of $\tan \beta$ and $A_0$ for which $\Delta \chi ^2 < 2.30$ or 5.99, respectively, and outside these contours there are no choices of $\tan \beta$ and $A_0$ that satisfy these conditions. In the figure, a low-mass “Crimea” region and a high-mass “Eurasia” region can be distinguished. The former consists of points in the stau-coannihilation region, and the latter includes points along rapid *H* / *A* annihilation funnels, and along the high-mass focus-point and stop-coannihilation strips we discuss in Sect. 6. We also show as a filled green star a representative best-fit point in the low-mass region, whose parameters are listed in Table 1. In the low-mass region, $g_\mu - 2$ makes a significantly smaller contribution to the global $\chi ^2$ function than in the high-mass region, although the CMSSM and related models could not by themselves resolve the discrepancy between the experimental measurement and the theoretical calculation within the Standard Model.^2^ We discuss later characteristics of points in the high-mass ‘Eurasia’ region: the $\chi ^2$ likelihood function is relatively flat across this region, and there is no well-defined best-fit point that is favoured strongly with respect to other points.

**Table 1:** Representative low-mass best-fit point found in a recent global CMSSM fit [33], using the ATLAS jets + $/ E_T$ constraint [3], and the combination of the LHCb [101] and CMS [102] constraints on $B_{s,d} \rightarrow \mu ^+ \mu ^-$ [103], and using FeynHiggs 2.10.0 [91] to calculate $m_h$

Model	Location	$m_0$	$m_{1/2}$	$A_0$	$\tan \beta$
		(GeV)	(GeV)	(GeV)	
CMSSM	Low-mass	670	1040	3440	21

In Fig. 1 we also show as a black line the 95 % CL exclusion contour in the $(m_0, m_{1/2})$ plane established by ATLAS searches for jets + $/ E_T$ events with $\sim$20/fb of data at 8 TeV [3]. This exclusion was derived within the CMSSM with $\tan \beta = 30$ and (in our sign convention) $A_0 = 2 \, m_0$, but studies have shown that the limit is relatively insensitive to the values of $\tan \beta$ and $A_0$ [32]. The ATLAS 95 % CLs contour intersects the 95 % CL contours found in the global fit, reflecting the importance of other observables in the global fit. For example, as already mentioned, $g_\mu - 2$ tends to favour relatively low values of $m_0$ and $m_{1/2}$. On the other hand, the measurement of $m_h$ tends to favour values of $m_0$ and $m_{1/2}$ beyond the ATLAS $/ E_T$ contour.

**Table 2:** Extrapolations of current LHC searches with $\sim$20/fb of luminosity at 8 TeV to higher energies and luminosities, assuming sensitivities to the same numbers of signal events. The first five rows of the table are possible 95 % CL exclusion sensitivities derived from searches for specific sparticle pair-production processes, as indicated, and the numbers correspond to the sparticle masses in GeV. The last two rows are for rays in the $(m_0, m_{1/2})$ plane, as indicated, and the numbers correspond to the possible 95 % CL exclusion limits on $m_0$ and $m_{1/2}$

	LHC			HE-LHC	FCC-hh
Search	8 TeV	14 TeV	14 TeV	33 TeV	100 TeV
Signature	20/fb	300/fb	3000/fb	3000/fb	3000/fb
$({\tilde{g}} \rightarrow b {\bar{b}} \chi )^2$ ($m_{\tilde{g}}$)	1300	2540	2990	6080	14700
${\tilde{t}}{\tilde{t}^*}$ ($m_{\tilde{t}}$)	650	1350	1740	3260	7020
$({\tilde{t}} \rightarrow c \chi )^2$ ($m_{\tilde{t}}$)	240	530	780	1320	2510
CMSSM	$(m_0, m_{1/2})$				
$m_0 = m_{1/2}$	(800, 800)	(1610, 1610)	(1860, 1860)	(4080, 4080)	(10,800, 10,800)
$m_0 = 2.5 m_{1/2}$	(1500, 600)	(2950, 1180)	(3390, 1360)	(7310, 2930)	(19,000, 7600)

We use a simple procedure to estimate the sensitivities of future collider searches exploiting the $/ E_T$ signature accompanied by jets (possibly *b*-tagged) and/or leptons at higher centre-of-mass energies and luminosities. We scale the 95 % CL exclusion or 5-$\sigma$ discovery contours of the searches at 8 TeV to different luminosity and energy scenarios by *assuming that the signal efficiency and background suppression of the current 8-TeV searches remain unchanged.* Maintaining the present performance of the searches is motivated by the ATLAS and CMS upgrade programmes, and is defined by both experiments as one of the main upgrade goals. The assumption was also used in several studies for Snowmass and ECFA (see e.g. [106, 107]) as well as to project collider limits for Dark Matter searches [108, 109]. It also forms the basis of the Collider Reach [110] tool, which reports dedicated studies showing good agreement between this extrapolation approach and results obtained from a full simulation.

We caution, however, that various effects could invalidate our assumption. For example, the signal-to-background ratio could vary with the centre-of-mass energy and with the number of pile-up events, which is correlated with the luminosity. Indeed, our extrapolation of the LHC sensitivities with 300/fb and 3000/fb of integrated luminosity at 14 TeV in the centre of mass is somewhat less conservative than ATLAS estimates of their exclusion sensitivities [111]. However, we have been able to verify that our simple assumption gives similar results to Snowmass estimates of the possible sensitivities of higher-energy colliders based on simplified model searches at the LHC with $\sim$20/fb of data at 8 TeV [106], and we consider our assumption a reasonable objective for future experimental analyses to target.

We consider in this paper the following LHC sparticle searches: searches for events with jets and missing transverse energy, $/ E_T$, possibly accompanied by leptons and with some jets *b*-tagged, dedicated searches for light stop squarks ${\tilde{t}} \rightarrow \chi + c$, and monojet searches. Using our simple assumption for a number of current LHC searches, we calculate cross sections at higher LHC centre-of-mass energies with Pythia 8 [112, 113], using as default the MSTW2008NLO parton distribution functions [114].^3^ We then require that the products of the integrated luminosity with the cross section be the same as for the 8 TeV LHC data. In this way, we extrapolate current LHC 95 % CLs exclusion limits to higher LHC energies and luminosities, as well as possible future colliders with 3000/fb at 33 and 100 TeV, as seen in Table 2.^4^

Figure 1 displays as purple and green lines, respectively, our extrapolations within the CMSSM of the current ATLAS 95 % CLs limit from searches for jets + $/ E_T$ events with $\sim$20/fb of data at 8 TeV to LHC searches at 14 TeV (LHC14) with 300/fb and 3000/fb of integrated luminosity. (We note that the ATLAS study [111] found that the 5-$\sigma$ discovery contour for 3000/fb almost coincides with the 95 % CLs exclusion contour for 300/fb.) Within the CMSSM, the ATLAS search for jets + $/ E_T$ events is the most sensitive for $m_0/m_{1/2} \le 2$, with other searches becoming more important at larger $m_0/m_{1/2}$. We return later to extrapolations of monojet searches and dedicated searches for light stop squarks, which are important for our studies of FCC-hh.

We see that the low-mass ‘Crimea’ region lies within the purple (95 % CLs exclusion with 300/fb or 5-$\sigma$ discovery with 3000/fb at 14 TeV) contour where $m_0 \le m_{1/2}$, whereas the high-mass ‘Eurasia’ region lies largely beyond the purple contour. Based on these comparisons between the extrapolated LHC sensitivity and current fits within the CMSSM, we have chosen for further study two scenarios for the outcome of the LHC searches with 3000/fb at 14 TeV.

- An ‘optimistic’ scenario in which the LHC discovers supersymmetry in the ‘Crimea’ region, and for definiteness we assume that its parameters coincide with those at the representative low-mass best-fit point in Table 1.
- A ‘pessimistic’ scenario in which the LHC discovers no evidence for supersymmetry, in which case the supersymmetry-breaking parameters must lie somewhere in ‘Eurasia’.

**Fig. 2:**
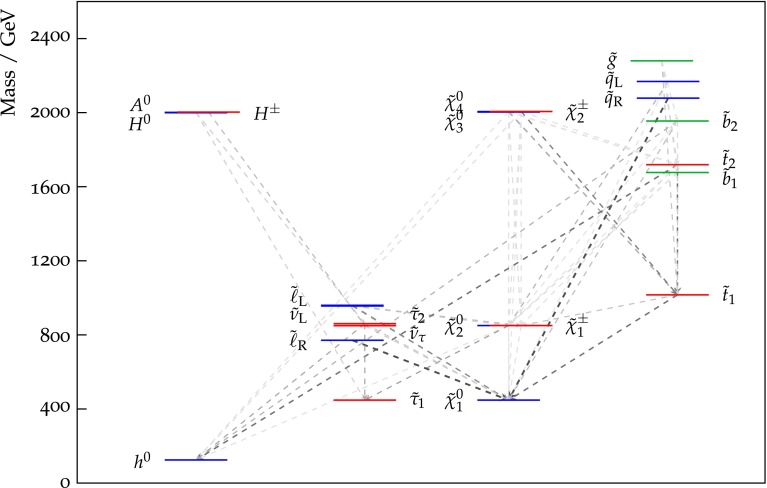
The spectrum at the best-fit point in the CMSSM [33], whose parameters are listed in Table 1. The magnitudes of the branching ratios for sparticle decays into different final-state particles are represented by the strengths of the *dashed lines* connecting them

**Fig. 3:**
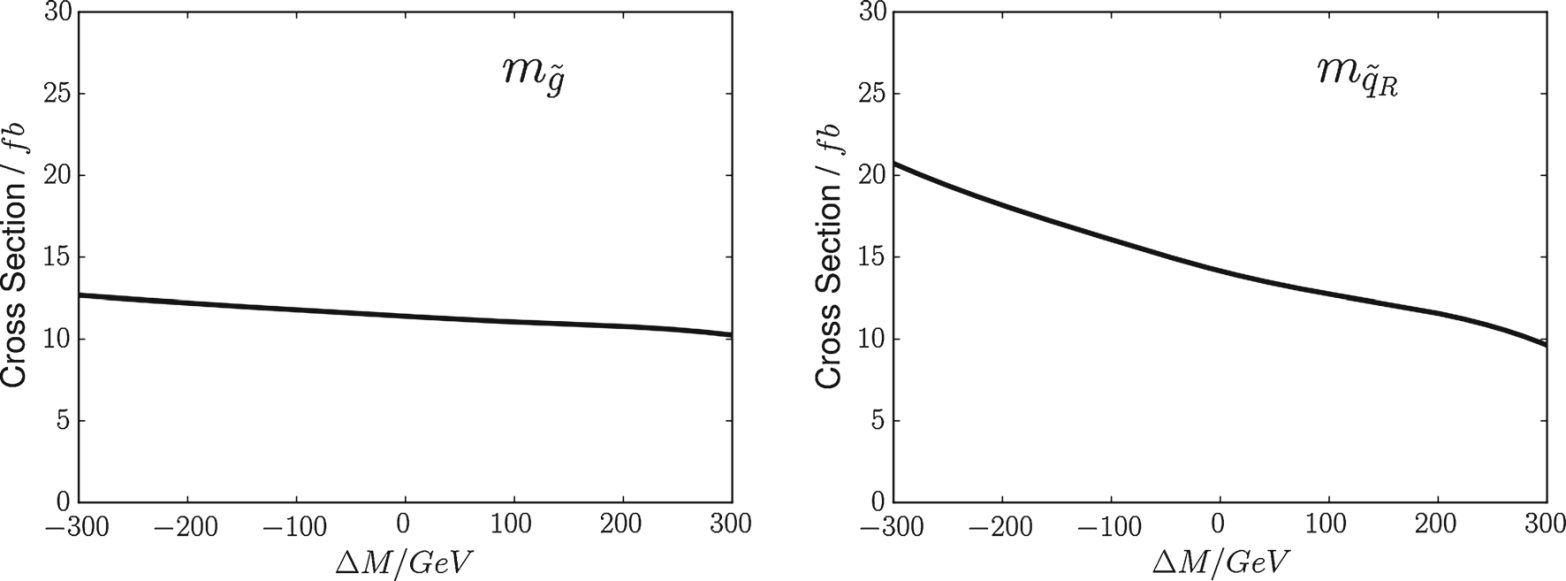
The sensitivities of the total sparticle cross section to $m_{\tilde{g}}$ (*left panel*) and $m_{\tilde{q}_R}$ (*right panel*), expressed as functions of the mass differences $\Delta M$ relative to the low-mass best-fit values in the CMSSM

The following sections contain discussions of the interplay between the various colliders in these scenarios.

## LHC measurements of supersymmetry in the optimistic scenario

Assuming that Nature is described by supersymmetry at the CMSSM low-mass best-fit point, the sparticle mass spectrum is determined, as illustrated in Fig. 2. The most relevant sparticles for searches at the LHC are those with the highest production cross sections, namely squarks and gluinos. At the best-fit point, the mass of a generic right-handed *u*, *d*, *s*, *c* or *b* squark is calculated to be $m_{\tilde{q}_R} \simeq 2080$ GeV, and the lighter stop squark has a mass $m_{\tilde{t}_1} \simeq 1020$ GeV. Also, $m_{\tilde{g}} \simeq 2280$ GeV and the lightest neutralino mass $m_\chi \simeq 450$ GeV. The lighter stau mass $m_{\tilde{\tau }_1}$ is only very slightly heavier: at the best-fit point and the rest of the low-mass region stau-$\chi$ coannihilation is responsible for bringing the relic density into the range allowed by cosmology. In the following we consider the possible LHC measurements of generic right-handed squarks ${\tilde{q}_R}$, gluinos ${\tilde{g}}$ and the lighter stop ${\tilde{t}_1}$, with either 300 or 3000/fb of luminosity at LHC14. We assume that experiments at the LHC discover supersymmetry with the mass spectrum characteristic of the best-fit point shown in Fig. 2, and we ask how accurately its parameters can be measured.

### Gluinos and squarks

We estimate first the potential resolution with which the gluino and squark masses could be measured. For this purpose, we consider three contributions to the determination of these model parameters: measurements of the total cross section, the distribution in the MT2 variable [116, 117], and the spectator jet energies in ${\tilde{g}} \rightarrow q + {\tilde{q}_R}$ decay. Figure 3 shows how the total cross section for strongly interacting particle production at LHC14 obtained from Pythia depends on the gluino mass $m_{\tilde{g}}$ (left panel) and the squark mass $m_{\tilde{q}_R}$ (right panel), expressed as functions of the mass differences $\Delta M$ relative to the low-mass best-fit values in the CMSSM.^5^ We see that the dependence of the cross section on $m_{\tilde{g}}$ is much weaker than that on $m_{\tilde{q}_R}$. In the following we combine the information that can be derived the cross section with that obtainable from an analysis using the MT2 variable.

**Fig. 4:**
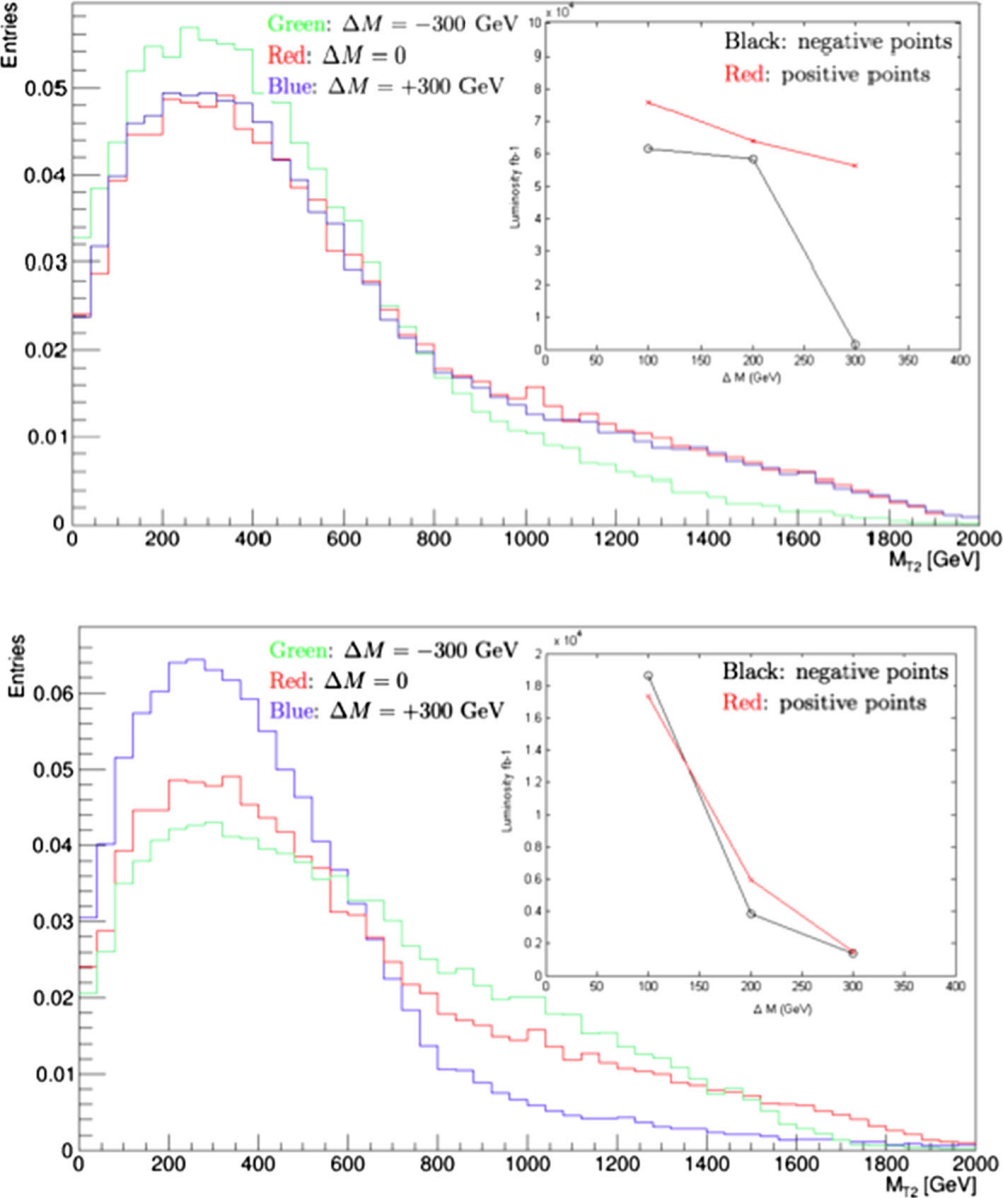
Simulations for 14-TeV collisions, using Pythia 8 [112, 113] and including Standard Model backgrounds, of the distributions in the MT2 variable for (*upper panel*) the nominal value of the gluino mass at the low-mass CMSSM best-fit point, $m_{\tilde{g}} \simeq 2280$ GeV (*blue histogram*), and gluino masses differing by $\pm 300$ GeV (*green* and *blue histograms*), and similarly for (*lower panel*) the nominal value of the squark mass $m_{\tilde{q}} \simeq 2080$ GeV and values $\pm 300$ GeV. In both cases, we fix the other sparticle masses to their nominal best-fit values, assuming in particular that the LSP mass $m_\chi = 450$ GeV. The *inserts* show the integrated luminosities at 14 TeV that would be required to distinguish at the 3-$\sigma$ level between the best fit and other models with the indicated mass shifts $\Delta M$

In order to assess how MT2 measurements could contribute to constraining the gluino and squark masses, we construct a set of MT2 templates for various values of these and the neutralino mass, and fit these templates to a simulation of the prospective MT2 distribution for the central best-fit values of the masses. For this analysis, we first matched the reconstructed jets from the Pythia output to the squarks, gluinos and neutralinos at the generator level. We then applied the same logic to construct MT2 as in experimental papers, treating the neutralinos as $/ E_T$ and the decay products of the squarks and gluinos as the jets. Thus, this approach does not consider combinatoric effects as could arise in a study that used a full detector simulation. We use the same kinematic specifications for the search regions as in the published 8 TeV search [3], and assume that the sensitivity remains the same for 14 TeV.^6^

Figure 4 displays prospective histograms of the MT2 distributions obtained from simulations using Pythia 8 [112, 113] and the MSTW2008NLO parton distribution functions [114] for different values of $m_{\tilde{g}}$ (upper panel), the right-handed squark mass $m_{\tilde{q}}$ (lower panel).^7^ In both cases, we compare the distribution for the nominal mass at the best-fit point with the corresponding distributions for values of the mass deviating from the nominal value by $\pm 300$ GeV, keeping the other sparticle masses fixed. In the gluino case, we see that the MT2 histogram for the nominal value $m_{\tilde{g}} = 2280$ GeV (in red) is very similar to that for the $- 300$ GeV choice (in blue), whereas the histogram for the $+ 300$ GeV choice is less similar. The reverse is true for the squark case (middle panel): here the nominal histogram for $m_{\tilde{q}_R} \simeq 2080$ GeV (red) is more similar to that for the $+ 300$ GeV choice (green), and less similar to that for the $-300$ GeV case (blue).

**Fig. 5:**
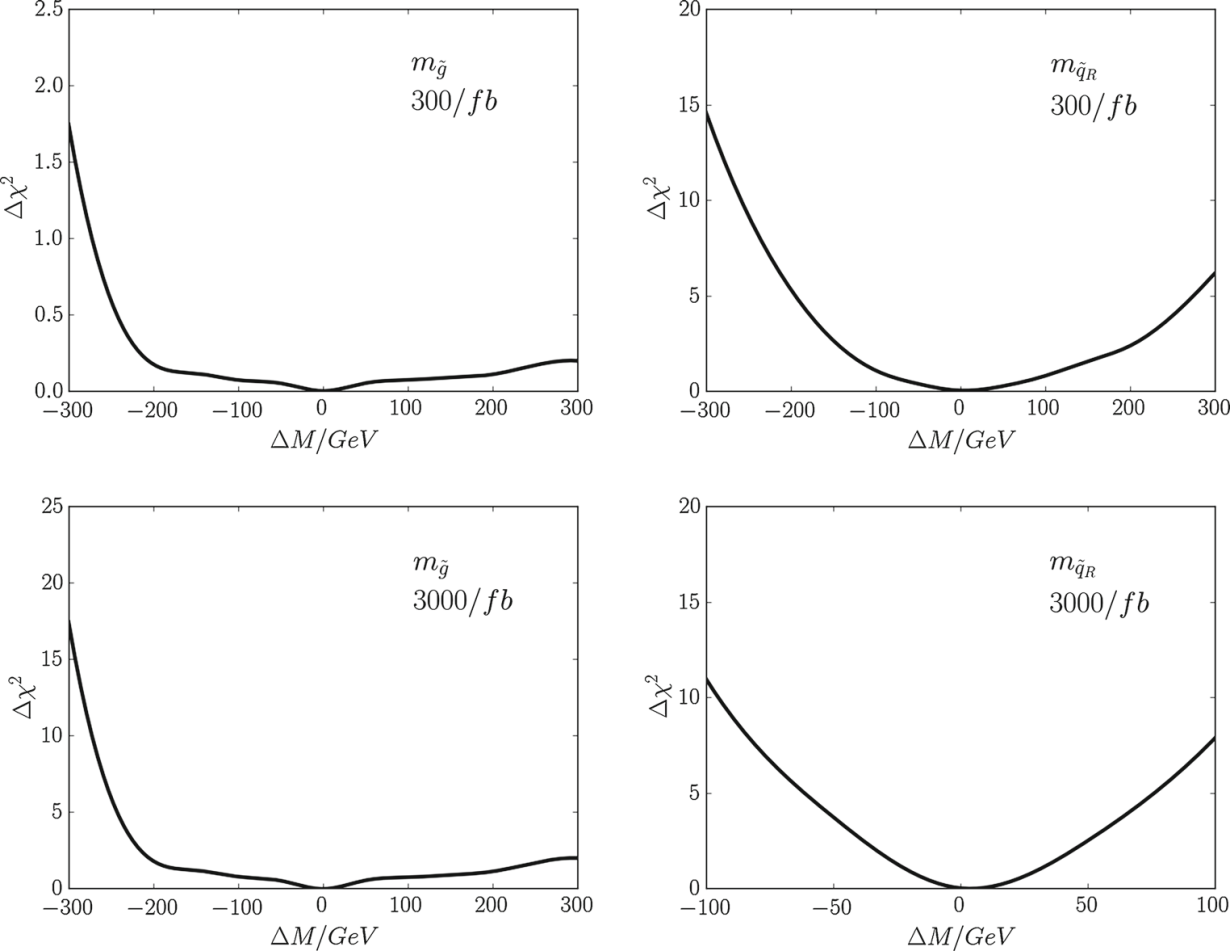
The $\chi ^2$ functions for $m_{\tilde{g}}$ (*left panels*) and $m_{\tilde{q}_R}$ (*right panels*), as estimated from cross-section and MT2 measurements with 300/fb (*upper panels*) and 3000/fb (*lower panels*)

The plots in Fig. 4 were obtained by recalculating the full Pythia output as the squark and gluino masses were varied around the best-fit CMSSM point. In some cases, the variation changed the ordering of the squark and gluino masses, leading to substantial changes in the MT2 distribution, e.g., in the $\Delta M = -300$ GeV case in the upper panel of Fig. 4 (green histogram) and in the $\Delta M = +300$ GeV case in the lower panel (blue histogram). The changes in the shapes of the MT2 distributions were less important when the mass ordering stayed the same. In addition, the variations in the shape of the MT2 distribution include the effects of changes in the relative production rates of ${\tilde{g}} {\tilde{g}}$, ${\tilde{g}} {\tilde{q}}$, ${\tilde{q}} {\tilde{q}}$ and ${\tilde{q}} {\tilde{\bar{q}}}$ final states arising from the mass variation.

In order to estimate the uncertainties in measurements of sparticle masses that could be possible at the LHC, we have performed fits to the simulated data for varying amounts of integrated luminosity. We use these to estimate the 68 % CL ranges of mass estimates obtainable with either 300 or 3000/fb of integrated luminosity. As seen in Fig. 4, the changes in the MT2 distributions for gluino and squark mass changes of $\pm 300$ GeV are quite different, so we do not expect symmetric Gaussian uncertainties, and we note that the same is true for the projected cross-section measurements shown in Fig. 3. Combining these with the MT2 measurements, we find the $\chi ^2$ distributions as functions of $m_{\tilde{g}}$ and $m_{\tilde{q}_R}$ shown in Fig. 5 in the left and right panels, respectively. The $\chi ^2$ functions are evaluated as

1 $$\begin{aligned} \chi ^2 (m) \; = \; \frac{1}{n} \sum _{i = 1}^n \left( \frac{(N_i (m) - N_i (\hat{m}))^2}{\sigma _i^2} \right) , \end{aligned}$$

where $\hat{m}$ is the nominal mass, the $N_i$ are numbers of events in the simulation and $\sigma _i$ is the statistical error in each bin for the assumed luminosity, and the sum over $i = 1, \ldots , n$ includes all the bins in the histograms added in quadrature. The upper row of panels is for 300/fb of integrated luminosity, and the lower row is for 3000/fb of integrated luminosity. On the basis of this analysis, we estimate the following fit uncertainties with 300/fb of data at 14 TeV:

2 $$\begin{aligned} 300/\mathrm{fb}: \; \; \Delta m_{\tilde{g}}= & {} (-270, + \cdots )~\mathrm{GeV} \, , \nonumber \\ \Delta m_{\tilde{q}_R}= & {} (-100, +110)~\mathrm{GeV}. \end{aligned}$$

where the $\ldots$ indicate that these measurements provide no useful upper limit on $m_{\tilde{g}}$, and with 3000/fb:

3 $$\begin{aligned} 3000/\mathrm{fb}: \; \; \Delta m_{\tilde{g}}= & {} (-110, +150)~\mathrm{GeV} \, , \nonumber \\ \Delta m_{\tilde{q}_R}= & {} (-30, +35)~\mathrm{GeV}. \end{aligned}$$

These uncertainties do not include a potential systematic effect from jet energy scale uncertainties. However, as we expect these to be at the level of 10 % or below, their overall impact is expected to be subdominant.

**Fig. 6:**
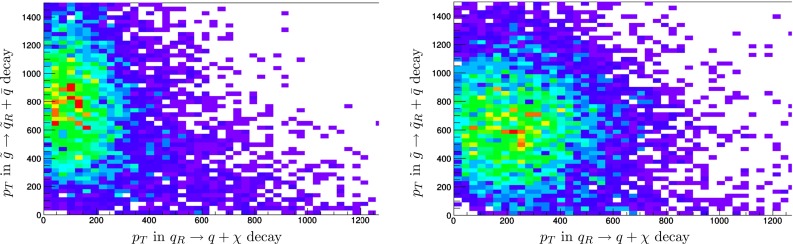
Scatter plots of the $p_T$ (in GeV) of the jet emitted in ${\tilde{q}_R} \rightarrow q + \chi$ decay (*horizontal axis*) and the jet emitted in ${\tilde{g}} \rightarrow {\tilde{q}_R} + {\bar{q}}$ decay (*vertical axis*) resulting from a simulation of gluino pair production at the LHC at 14 TeV. *Left panel* for the best-fit ${\tilde{g}}$ and ${\tilde{q}_R}$ masses. *Right panel* for the best-fit values of $m_{\tilde{g}}$ but with $m_{\tilde{q}_R}$ reduced by 300 GeV

**Fig. 7:**
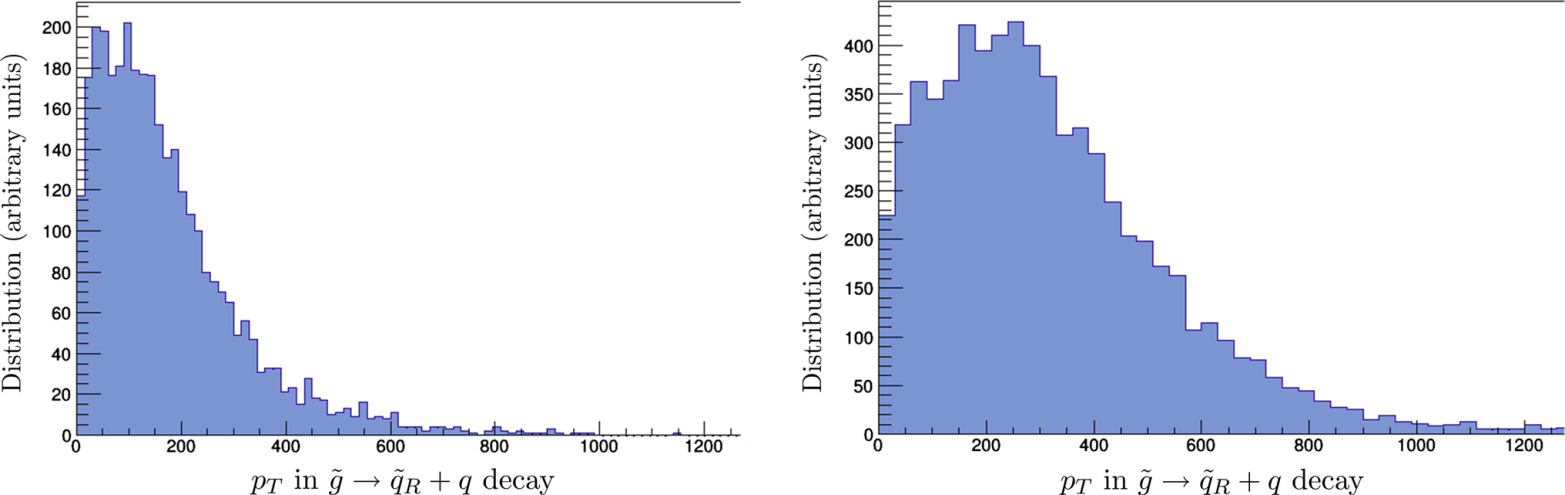
Simulations of the distributions of the quark $p_T$ (in GeV) from ${\tilde{g}}$ pair production at the LHC at 14 TeV followed by ${\tilde{g}} \rightarrow {\tilde{q}_R} + q$ decays. *Left panel* for the best-fit ${\tilde{g}}$ and ${\tilde{q}_R}$ masses, and (*right panel*) for the same value of $m_{\tilde{g}}$ but with $m_{\tilde{q}_R}$ reduced by 300 GeV

The upper and lower panels of Fig. 4 show that the mass difference $m_{\tilde{g}} - m_{\tilde{q}_R}$ is poorly constrained by the MT2 measurement, and this is reflected in the asymmetric $\chi ^2$ distributions seen in Fig. 5. However, there are many other possible measurements at the LHC. In particular, we have considered the extra information that could be obtained from measurements of the (relatively) soft jet emitted in the decay ${\tilde{g}} \rightarrow {\tilde{q}_R} + {\bar{q}}$, which would be monochromatic in the gluino rest frame. Figure 6 displays scatter plots of the $p_T$ of the jet emitted in ${\tilde{q}_R} \rightarrow q + \chi$ decay (horizontal axis) and the jet emitted in ${\tilde{g}} \rightarrow {\tilde{q}_R} + {\bar{q}}$ decay (vertical axis) based on simulations of ${\tilde{g}}$ pair production. The left panel is for the best-fit values of $m_{\tilde{g}}$ and $m_{\tilde{q}_R}$, and the right panel is for the same value of $m_{\tilde{g}}$ but with $m_{\tilde{q}_R}$ reduced by 300 GeV: the plots are clearly distinct.

Figure 7 displays the spectrum of the ‘soft’ jet in the same two cases: in the left panel with the best-fit ${\tilde{g}}$ and ${\tilde{q}_R}$, and in the right panel with the same value of $m_{\tilde{g}}$ but with $m_{\tilde{q}_R}$ reduced by 300 GeV. These can clearly be distinguished with a high degree of confidence. We do not display the corresponding distribution with $m_{\tilde{q}_R}$ increased by 300 GeV, since in this case the ${\tilde{q}_R}$ is heavier than the ${\tilde{g}}$ and there is no ‘monochromatic’ supplementary jet in gluino decay.

**Fig. 8:**
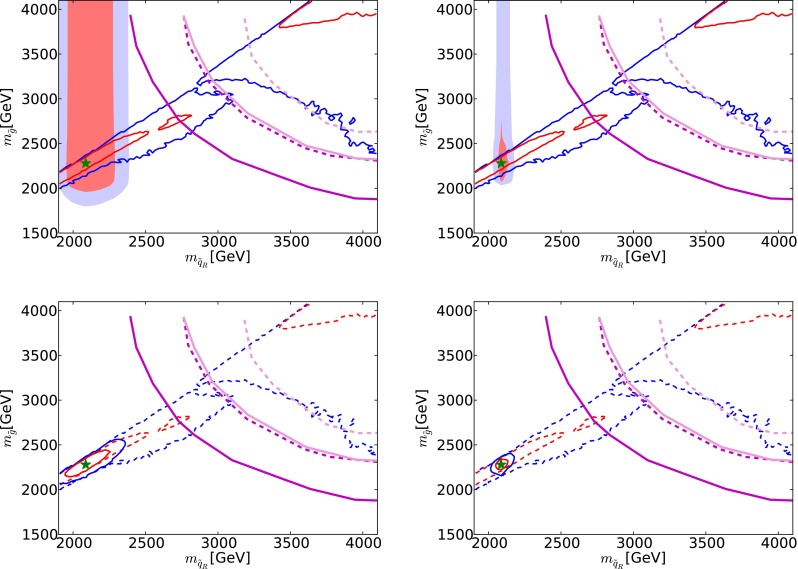
The *upper panels* show the 68 and 95 % CL regions (*shaded pink* and *blue*, respectively) in the $(m_{\tilde{q}_R}, m_{\tilde{g}})$ planes obtained from cross-section, MT2 and supplementary jet measurements at LHC14 with 300/fb (*left panel*) and 3000/fb (*right panel*). These regions are superposed on the best-fit point (*green star*) and the 68 and 95 % CL regions found in a recent global fit to the CMSSM [33] (*solid red* and *blue lines*), and the *magenta lines* show the prospective capabilities of the LHC to exclude at the 95 % CL (*dashed*) supersymmetry or discover it at the 5-$\sigma$ level (*solid*) with 300/fb or 3000/fb (*darker* and *lighter lines*). In the *lower panels* we show as *solid red* and *blue lines* the results of fits combining the LHC measurements with the recent global fit (here shown as *dashed lines*)

We assume that the jet energy in ${\tilde{g}} \rightarrow {\tilde{q}_R} + {\bar{q}}$ decay can be measured with an accuracy of 50 GeV. This information can then be combined with the cross-section and MT2 distribution discussed earlier to estimate 68 and 95 % CL regions in the $(m_{\tilde{q}_R}, m_{\tilde{g}})$ plane. These are shown shaded pink and blue, respectively, in Fig. 8 for 300/fb of integrated luminosity (upper left panel) and for 3000/fb of integrated luminosity (upper right panel). As in Fig. 1, the low-mass portions of the solid red and blue contours outline the Crimea region and the high-mass portions correspond to the Eurasia region. Finally, the solid [dashed] magenta lines (darker and lighter) show the 5-$\sigma$ discovery (95 % CL exclusion) reaches of the LHC with 300 (3000)/fb. The lower panels of Fig. 8 show as solid red and blue lines the 68 and 95 % CL contours from fits combining the prospective LHC measurements with the recent global fit [33] (whose CL contours are displayed as dashed lines in these panels).

**Fig. 9:**
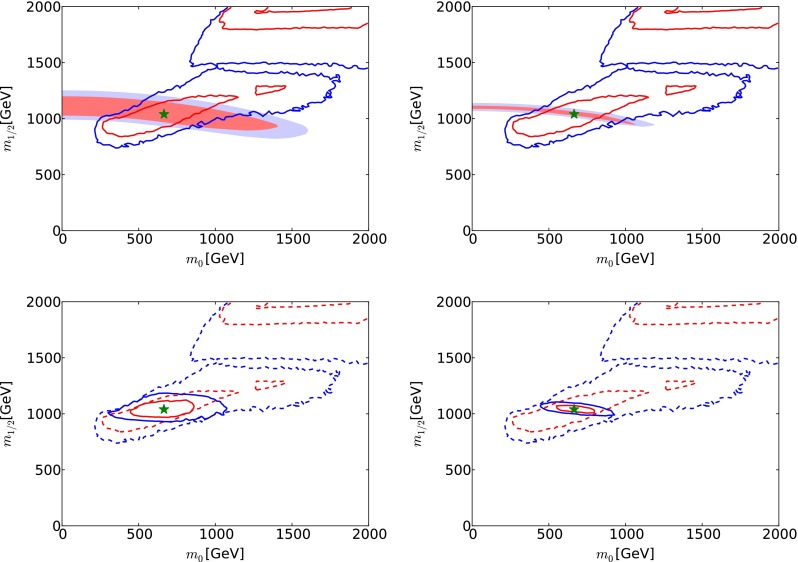
The *upper panels* show the 68 and 95 % CL regions (*shaded pink* and *blue*, respectively) in the $(m_0, m_{1/2})$ planes obtained from cross-section, MT2 and supplementary jet measurements at LHC14 with 300/fb (*left panel*) and 3000/fb (*right panel*). These regions are superposed on the 68 and 95 % CL regions found in a recent global fit to the CMSSM [33] (*red* and *blue lines*). In the *lower panels* we show as *solid lines* the results of fits combining the LHC measurements with this global fit (here shown as *dashed lines*)

These prospective measurements can be projected onto the $(m_0, m_{1/2})$ plane of the CMSSM, as seen in the upper panels of Fig. 9, also for 300/fb of integrated luminosity (upper left panel) and 3000/fb of integrated luminosity (upper right panel). Formally, the corresponding numerical 68 % CL uncertainties are

4 $$\begin{aligned} 300/\mathrm{fb}: \; \; \Delta m_0= & {} (-670, +620)~\mathrm{GeV} \, , \nonumber \\ 3000/\mathrm{fb}: \; \; \Delta m_0= & {} (-670, +220)~\mathrm{GeV}, \end{aligned}$$

and

5 $$\begin{aligned} 300/\mathrm{fb}: \; \; \Delta m_{1/2}= & {} (-140, +100)~\mathrm{GeV} \, , \nonumber \\ 3000/\mathrm{fb}: \; \; \Delta m_{1/2}= & {} (-90, +20)~\mathrm{GeV}. \end{aligned}$$

The upper panels of Fig. 9 also show that these numbers imply non-trivial correlations between $m_0$ and $m_{1/2}$. They also show, as solid red and blue lines, respectively, the boundaries of the 68 and 95 % CL regions found in the recent global analysis of current data [33]. We see that the prospective future LHC measurements could provide information that would be complementary to this global fit [33].^8^

The lower panels of Fig. 9 show the results (solid red and blue lines) of combining these LHC measurements with the recent global fit [33] (dashed red and blue lines). We see that the LHC measurements would reduce substantially the sizes of the 68 and 95 % CL regions already with 300/fb, and that the prospective 3000/fb measurements would be particularly powerful in this regard.

### Stop measurements

**Fig. 10:**
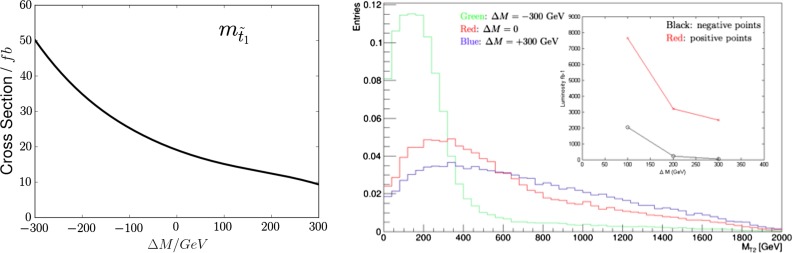
*Left panel* the sensitivity of the total stop pair-production cross section to $m_{\tilde{t}_1}$. *Right panel* Simulations for 14-TeV collisions of the distributions in the MT2 variable for the nominal value of the lighter stop mass $m_{\tilde{t}_1} = 1020$ GeV and values $\pm 300$ GeV, with the other sparticle masses fixed to their nominal best-fit values. The *insert* shows the integrated luminosities at 14 TeV that would be required to distinguish at the 3-$\sigma$ level between the best fit and other models with the indicated mass shifts $\Delta M$ relative to the value at the low-mass best-fit point

We have also considered the possible accuracy in measuring $m_{\tilde{t}_1}$ via ${\tilde{t}_1} + \overline{\tilde{t}_1}$ production at the LHC at 14 TeV. The left panel of Fig. 10 shows the sensitivity of the total stop pair-production cross section to $m_{\tilde{t}_1}$: we see that over the displayed range it is greater than those to the $m_{\tilde{g}}$ and $m_{\tilde{q}_R}$, which were shown in Fig. 3. The right panel of Fig. 10 shows histograms of MT2 for the nominal mass $m_{\tilde{t}_1} \simeq 1020$ GeV at the representative low-mass best-fit point and for choices differing by $\pm 300$ GeV. These cases are quite distinct, as is also seen in the inset, which displays the luminosities required for 3-$\sigma$ discrimination between the nominal value of $m_{\tilde{t}_1}$ and selected larger or smaller values.

**Fig. 11:**
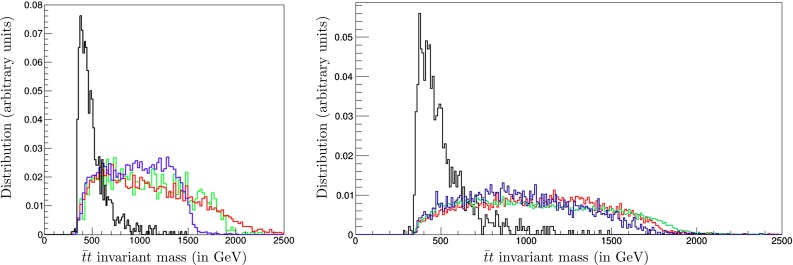
The unit-normalised $t {\bar{t}}$ invariant-mass distribution resulting from a simulation of ${\tilde{t}_1} + \overline{\tilde{t}_1}$ production at the LHC at 14 TeV. *Left panel* for the best-fit ${\tilde{g}}$ and ${\tilde{t}_1}$ masses of 2280 and 1020 GeV (*green histogram*), compared with the Standard Model background (*black histogram*) and simulations with ${\tilde{g}}$ masses 300 GeV above (*red histogram*) and 300 GeV below (*blue histogram*) the nominal value of $m_{\tilde{g}}$. *Right panel* similarly for the best-fit ${\tilde{g}}$ and ${\tilde{t}_1}$ masses (*green histogram*), compared with the Standard Model background (*black histogram*) and simulations with ${\tilde{t}_1}$ masses 300 GeV above (*red histogram*) and 300 GeV below (*blue histogram*) the nominal value of $m_{\tilde{t}_1}$

The left panel of Fig. 11 displays the shape of the unit-normalised $t {\bar{t}}$ invariant-mass distribution resulting from a simulation of such events using Pythia 8 [112, 113] and the MSTW2008NLO parton distribution functions [114], produced with the nominal CMSSM best-fit values of $m_{\tilde{t}_1} = 1020$ GeV and $m_{\tilde{g}} = 2280$ GeV (green histogram), compared with the Standard Model background (black histogram), which is sharply peaked at low invariant masses close to the $t {\bar{t}}$ threshold. Also shown in Fig. 11 are the invariant-mass distributions for ${\tilde{g}}$ masses 300 GeV above (red histogram) and 300 GeV below (blue histogram) the nominal value of $m_{\tilde{t}_1}$. As expected, the higher (lower) mass gives a longer (shorter) tail in the invariant-mass distribution. On the other hand, as we see in the right panel of Fig. 11 that the invariant ${\tilde{t}_1} + \overline{\tilde{t}_1}$ mass distribution in ${\tilde{g}}$ decays is almost independent of $m_{\tilde{t}_1}$ for fixed $m_{\tilde{g}}$.

**Fig. 12:**
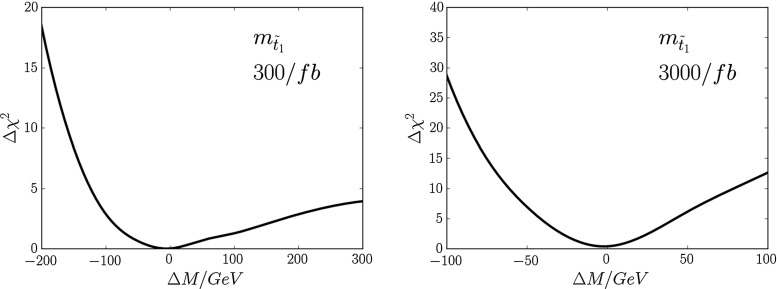
The $\chi ^2$ functions for $m_{\tilde{t}_1}$ estimated from LHC14 measurements with 300/fb (*left panel*) and 3000/fb (*right panel*)

Combining the cross-section, MT2 and $t {\bar{t}}$ invariant-mass measurements, we find the $\chi ^2$ distributions as functions of $m_{\tilde{t}_1}$ shown in Fig. 12. The left panel is for 300/fb of integrated luminosity, and the right panel is for 3000/fb of integrated luminosity. We find the following fit uncertainties with 300/fb or 3000/fb of data at 14 TeV:

6 $$\begin{aligned} 300/\mathrm{fb}: \; \; \Delta m_{\tilde{t}_1}= & {} (-30, +50)~\mathrm{GeV} \, , \nonumber \\ 3000/\mathrm{fb}: \; \; \Delta m_{\tilde{t}_1}= & {} (-10, +15)~\mathrm{GeV}. \end{aligned}$$

As in the previous cases, these uncertainties should be convoluted with a systematic jet energy scale uncertainty of $\sim$10 %.

The uncertainties (6) may be used to estimate the corresponding uncertainties in the trilinear soft supersymmetry-breaking parameter $A_0$, by comparing the stop mass (which is very sensitive to $A_0$) with the squark and gluino masses (which are insensitive to $A_0$). The effect of marginalising over the latter masses can be incorporated by assuming that $m_0$ and $m_{1/2}$ have their best-fit values, as is also the case for $\tan \beta$. In estimating the uncertainty in $A_0$, we incorporate the correlation between $A_0$ and $\mu$ that is imposed by the electroweak vacuum conditions within the CMSSM, finding

7 $$\begin{aligned} 300/\mathrm{fb}: \; \; \Delta A_0= & {} (+80, -150)~\mathrm{GeV} \, , \nonumber \\ 3000/\mathrm{fb}: \; \; \Delta A_0= & {} (+30, -40)~\mathrm{GeV}. \end{aligned}$$

We emphasise again that these uncertainties do not take into account the jet energy scale uncertainty, which we expect to be subdominant.

As a final point in this section, we comment on the magnitudes of some of the branching ratios for sparticle decays that are represented by dashed lines in Fig. 2. The earlier analysis of ${\tilde{g}} \rightarrow {\tilde{q}} + {\bar{q}}$ decays exploits the fact that this decay mode is dominant if $m_{\tilde{g}} > m_{\tilde{q}_R}$, as reflected in the boldness of the dashed line connecting the ${\tilde{g}}$ and ${\tilde{q}_R}$ states. We draw attention to the decay ${\tilde{t}_2} \rightarrow {\tilde{t}_1} + h$, which is also dominant, having a branching ratio of 77 % represented also by a bold dashed line. This implies that about 50 % of ${\tilde{t}_2} \overline{\tilde{t}_2}$ events would contain, in addition to a $t {\bar{t}}$ pair, a pair of high-$p_T$ Higgs bosons and substantial missing transverse energy. Typical boost factors for the Higgs bosons would be $\sim$5. A detailed exploration of this experimental signature lies beyond the scope of this paper.

## $e^+ e^-$ probes of supersymmetry in the optimistic scenario

In the low-mass ‘optimistic’ CMSSM scenario there would be interesting opportunities for both direct and indirect precision probes of supersymmetry at an $e^+ e^-$ collider, which we now explore.

### Direct sparticle-pair production

**Fig. 13:**
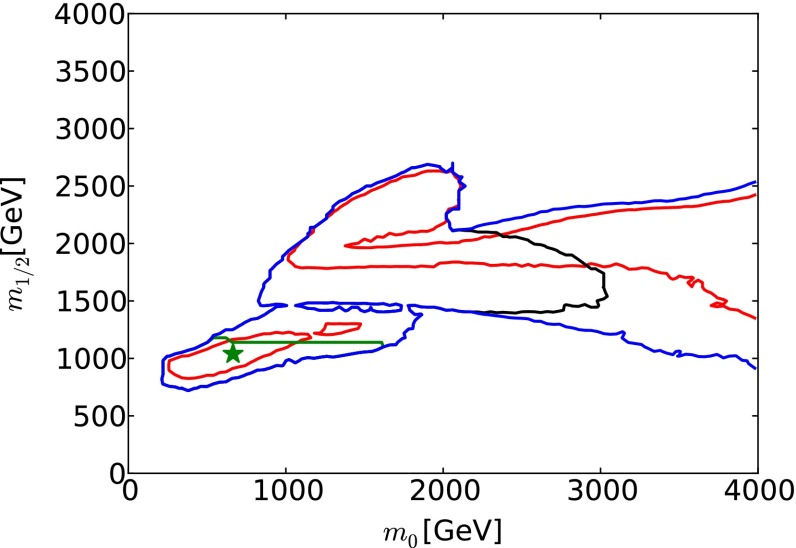
Contours where it is possible to attain at the 95 % CL $m_{\tilde{\tau }_1} = 500 \, (1500)$ GeV, indicated by *solid green* (*black*) *lines*, are overlaid on the $(m_0, m_{1/2})$ plane in the CMSSM, with the same CL contours and best-fit point from a global fit [33] as displayed previously in Fig. 1

The most direct possibility would be pair production and measurement of electroweakly interacting sparticles. The next-to-lightest supersymmetric particle (NLSP) is expected, in generic regions of the CMSSM parameter space, to be the lighter stau slepton ${\tilde{\tau }_1}$. Accordingly, Fig. 13 displays, superimposed on the same CMSSM $(m_0, m_{1/2})$ plane discussed previously, contours showing where it is possible at the 95 % CL to attain $m_{\tilde{\tau }_1} = 500$ GeV (green), the largest mass that could be pair-produced with an $E_{CM} = 1$ TeV linear collider, and 1500 GeV (black), the largest mass that could be pair-produced with an $E_{CM} = 3$-TeV linear collider such as CLIC. These contours are restricted to the regions within the 68 and 95 % CL regions found in the recent global fit [33], where the CMSSM parameter space is well sampled. We see that the $m_{\tilde{\tau }_1} = 500$ GeV line crosses the ‘Crimea’ region, whereas the $m_{\tilde{\tau }_1} = 1500$ GeV lines reach deep into the ‘Eurasia’ region. In particular, the low-mass best-fit point in the CMSSM lies within the $m_{\tilde{\tau }_1} = 500$ GeV reach of a 1-TeV $e^+ e^-$ collider. In the low-mass ‘Crimea’ region, the cold dark matter density is brought into the range acceptable to cosmology by coannihilation with the stau, so the $m_{\tilde{\tau }_1} \le 500$ GeV contour has $m_{1/2}$ almost constant. On the other hand, in the ‘Eurasia’ region other mechanisms such as neutralino annihilation via direct-channel heavy Higgs poles come into play, and the $m_{\tilde{\tau }_1} \le 1500$ GeV contour has a more complicated shape.

Within the specific CMSSM model studied, a 500-GeV $e^+ e^-$ collider would very likely be unable to detect directly any supersymmetric particles. This is because the contour for $m_{\tilde{\tau }_1} = 250$ GeV, the largest mass that could be pair-produced with an $E_{CM} = 500$-GeV linear collider, would lie at $m_{1/2} \lesssim 600$ GeV, which is outside the 95 % CL contour in the $(m_0, m_{1/2})$ plane shown in Fig. 13. A similar conclusion could be drawn from the lower right panels of Figs. 5 and 13 of [33], where we see that $\Delta \chi ^2 > 9$ for $m_{\tilde{\tau }_1} \le 250$ GeV.^9^

**Fig. 14:**
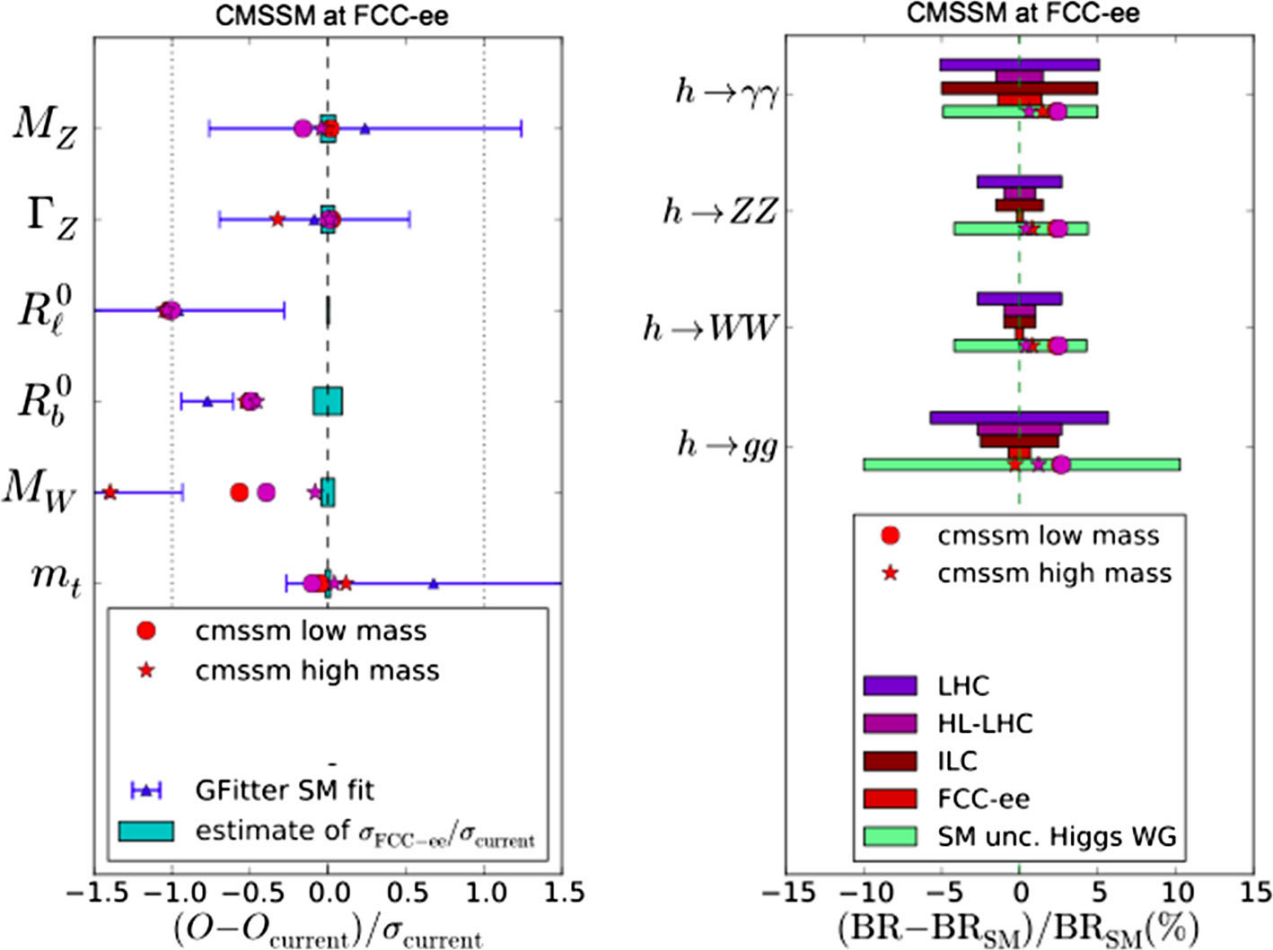
The *left panel* compares current measurements of electroweak precision observables (EWPOs) taken from a Standard Model fit [118] (*blue*, with *error bars*), predictions at low- and high-mass best-fit points in the CMSSM [33] (*red* and *purple symbols*) and prospective FCC-ee (TLEP) experimental errors [65] (*turquoise bars*). The *right panel* compares prospective measurements of Higgs branching ratios at future colliders, low- and high-mass CMSSM predictions (*red* and *purple symbols*) and the current uncertainties within the Standard Model (*turquoise bars*)

If slepton–antislepton pair production is accessible at an $e^+ e^-$ collider, many very precise direct measurements become possible. Two benchmark supersymmetric scenarios were analysed in [59], and the prospective accuracies for sparticle mass measurements were assessed. In one of these scenarios (P1), the slepton mass spectrum was very similar to that at the low-mass CMSSM best-fit point (see Table 1; Fig. 2), with masses between 1000 and 1100 GeV. The ${\tilde{\chi }_1^\pm }$ and ${\tilde{\chi }_1^0}$ masses in scenario P1 were somewhat lower than in the low-mass best-fit CMSSM spectrum, whereas the ${\tilde{\chi }_2^0}$ mass was again very similar. Based on simulations of 2/ab of CLIC data at 3 TeV, the following uncertainties in the sparticle masses were estimated:

8 $$\begin{aligned}&\Delta m_{\tilde{e}_R} = 2.9 \, \mathrm{GeV}, \; \Delta m_{\tilde{\chi }_1^0} \; = \; 4.6 \, \mathrm{GeV}, \nonumber \\&\Delta m_{\tilde{\chi }_1^\pm } \; = \; 3.6 \, \mathrm{GeV}. \end{aligned}$$

The measurement uncertainty in $m_{\tilde{\chi }_1^0}$ can be converted directly into the corresponding uncertainty in $m_{1/2}$:

9 $$\begin{aligned} \Delta m_{1/2} \; = \; 11 \, \mathrm{GeV}. \end{aligned}$$

Combining this uncertainty with the uncertainty in the $m_{\tilde{e}_R}$ measurement, one finds

10 $$\begin{aligned} \Delta m_0 \; = \; 4 \, \mathrm{GeV}. \end{aligned}$$

As discussed in [59] and [60], many precision measurements of supersymmetric particle masses and other properties would be possible at CLIC point, including tests of the universality hypothesis of the CMSSM. However, it is already clear that CLIC could provide exceptional precision in the determination of CMSSM model parameters, if Nature is described by a model in the Crimea region. Moreover, the comparison between the CLIC determinations of the CMSSM parameters with those from the LHC discussed earlier would enable non-trivial checks to be made of the consistency of the CMSSM assumption of universal input soft supersymmetry-breaking parameters.

### Electroweak precision observables

It is also possible to obtain indirect information about supersymmetric models from electroweak precision observables (EWPOs), similar in principle to the information about $m_t$ and $m_H$ obtained previously from precision measurements at LEP and the SLC [61].

**Fig. 15:**
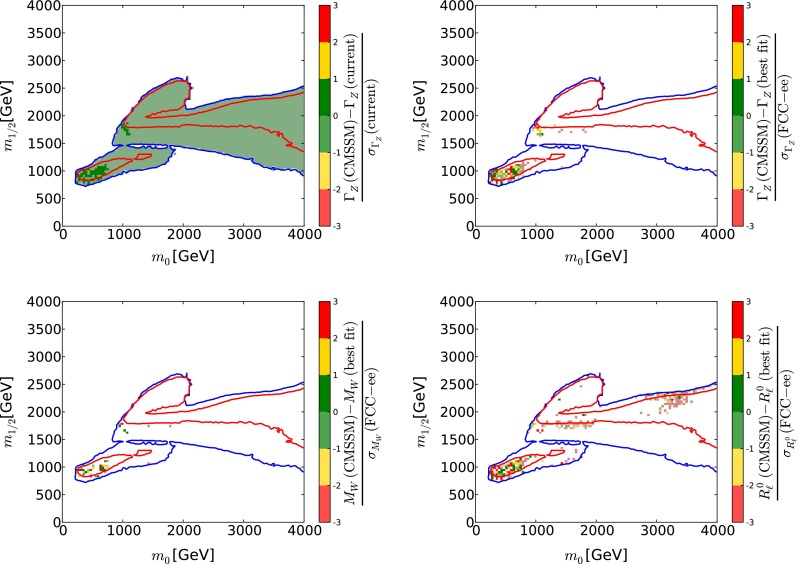
The present measurement of $\varGamma _Z$ (*upper left panel*) [61], and prospective FCC-ee (TLEP) measurements [65] of $\varGamma _Z$ (*upper right*), $M_W$ (*lower left*) and $R_\ell$ (*lower right*) are superposed on the preferred region of the $(m_0, m_{1/2})$ plane in the CMSSM [33] shown previously in Fig. 1. The *colours* represent deviations from the present central value in units of the present LHC experimental error (*upper left panel*), and the deviations from the values at the low-mass best-fit point of the values at other points in the $(m_0, m_{1/2})$ plane in units of the estimated future FCC-ee (TLEP) experimental errors (*other panels*)

The left panel of Fig. 14 displays as blue points with error bars the central values and 1-$\sigma$ uncertainties of several such observables, as calculated in a recent global fit [118], compared with their values and current individual experimental uncertainties in the Standard Model. Also shown (without theoretical uncertainties) are the values of these observables calculated at the representative low- and high-mass best-fit points in the CMSSM found in [33]. As is apparent from the left panel of Fig. 14 and the upper left panel of Fig. 15, the current experimental error in the measurement of $\varGamma _Z$ is too large to provide much information about supersymmetric model parameters. The entire region of the CMSSM $(m_0, m_{1/2})$ plane currently allowed at the 95 % CL according to the global fit [33] is compatible with the current measurement of $\varGamma _Z$ at the 1-$\sigma$ level [61]. However, also shown in the left panel of Fig. 14, as turquoise bars, are the prospective experimental errors in measurements at FCC-ee (TLEP) (neglecting theoretical uncertainties) [65], normalised relative to the current experimental errors. It is clear that, for $\varGamma _Z$ and many other electroweak precision observables, the prospective FCC-ee (TLEP) uncertainties are sufficiently small to be very sensitive to deviations from their Standard Model values and capable of constraining supersymmetric scenarios.

The right panel of Fig. 14 makes a similar point for precision Higgs observables, by comparing the estimated precisions of measurements at the LHC, the ILC and FCC-ee (TLEP) [65, 119] (shown as colour-coded horizontal bars) with the deviations of the observables from their Standard Model values that are calculated for the low- and high-mass CMSSM best-fit points [33]. It is clear that FCC-ee (TLEP) has the best ability to distinguish these models from the Standard Model, as we discuss in more detail later.

As a first example of the possible utility of the precision electroweak measurements possible with FCC-ee (TLEP), we consider the optimistic scenario in which supersymmetry is within the LHC discovery range and assume, for definiteness, that the model parameters correspond to the current best-fit low-mass point in the CMSSM. We see in the upper left panel of Fig. 15 that, as already commented, the current experimental uncertainty in $\varGamma _Z$, namely $\Delta \varGamma _Z = 2.3$ MeV [61], is too large to provide significant information about CMSSM model parameters within the 95 % CL regions displayed in Fig. 1. For this reason, all of the 95 % CL region in the upper left panel of Fig. 1 is shaded green, since it lies within one current standard deviation of the present measurement. On the other hand, we see in the upper right panel of Fig. 15 that the prospective experimental uncertainty at FCC-ee (TLEP), namely $\Delta \varGamma _Z = 0.1$ MeV [65], is far smaller than the variation in $\varGamma _Z$ across even the CMSSM 68 % CL Crimea region. For this reason, much of the 68 and 95 % CL regions in this panel of Fig. 1 are unshaded, since they lie more than three current standard deviations away from the prospective measurement. The same holds for other electroweak precision observables such as $M_W$ (prospective experimental uncertainty 0.5 MeV [65], lower left panel of Fig. 15), $R_\ell$ (prospective experimental uncertainty $5 \times 10^{-5}$ [65], lower right panel of Fig. 15) and others not shown.

**Fig. 16:**
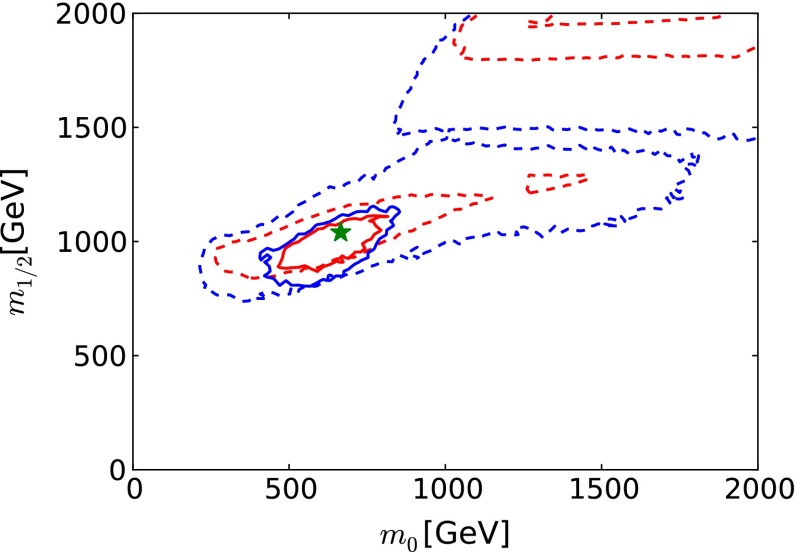
The prospective $\Delta \chi ^2 = 2.30$ (68 % CL) and $\Delta \chi ^2 = 5.99$ (95 % CL) contours (*solid red* and *blue lines*, respectively) in the $(m_0, m_{1/2})$ plane for the CMSSM (with the present 68 and 95 % CL contours shown as *dashed red* and *blue lines*, respectively), assuming that the electroweak precision observables are measured at FCC-ee (TLEP) to have the same central values as at the current low-mass CMSSM best-fit point [33] (shown as the *filled green star*), and neglecting inevitable improvements in other constraints on the supersymmetric models

We have made a crude estimate of the impact on the recent global fit to the CMSSM parameters of these FCC-ee (TLEP) electroweak measurements, neglecting the inevitable improvements in flavour, dark matter and Higgs observables, and setting aside the direct measurements of sparticle masses possible at the LHC following discovery in this optimistic scenario. As we see in Fig. 16, the electroweak precision measurements would, by themselves, provide very tight constraints on the CMSSM parameters $m_0$ and $m_{1/2}$.

**Fig. 17:**
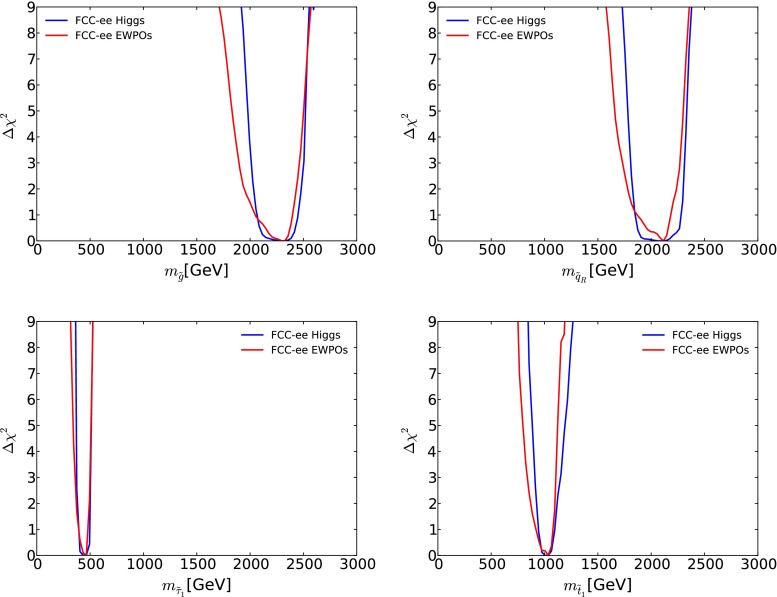
The one-dimensional $\Delta \chi ^2$ profile likelihood functions for the gluino mass $m_{\tilde{g}}$ (*upper left panel*), the generic first- and second-generation squark mass $m_{\tilde{q}}$ (*upper right panel*), the lighter stop squark mass (*lower left panel*) and the lighter stau mass (*lower right panel*), as obtained using prospective FCC-ee (TLEP) precision electroweak measurements (*solid red lines*) and Higgs measurements (*solid blue lines*) [65] with the same central values as the low-mass best-fit CMSSM point [33], neglecting the inevitable improvements in other constraints on the supersymmetric models

**Fig. 18:**
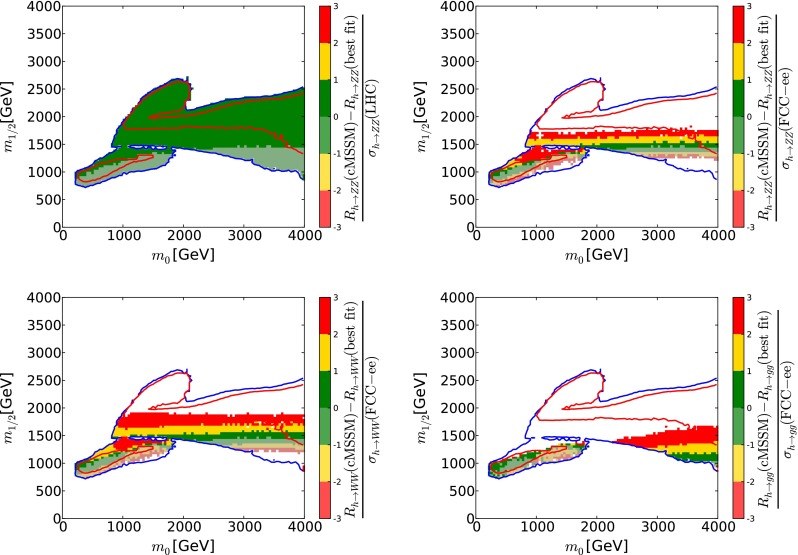
The present measurement of BR($H \rightarrow ZZ$) (*upper left panel*), and prospective FCC-ee (TLEP) measurements [65] of BR($H \rightarrow ZZ$) (*upper right*), BR($H \rightarrow \gamma \gamma$) (*lower left*) and BR($H \rightarrow gg$) (*lower right*) are superposed on the $(m_0, m_{1/2})$ plane in the CMSSM shown previously in Fig. 1. The *colours* represent deviations from the present central value in units of the present LHC experimental error (*upper left panel*), and the deviations from the values at the low-mass best-fit CMSSM point [33] of the values at other points in the $(m_0, m_{1/2})$ plane in units of the estimated future FCC-ee (TLEP) experimental errors [65] (*other panels*)

After inclusion of the FCC-ee (TLEP) measurements [65] in this optimistic scenario, only a small part of the low-mass ‘Crimea’ region is allowed at the 68 or 95 % CL, as seen in Fig. 16. The impact of the FCC-ee (TLEP) measurements may be translated into the one-dimensional likelihood functions for various sparticle masses, shown as solid red lines in Fig. 17.^10^ We see that $m_{\tilde{g}}$, $m_{\tilde{q}}$, $m_{\tilde{\tau }_1}$ and $m_{\tilde{t}_1}$ could be estimated with interesting accuracy on the basis of FCC-ee (TLEP):

11 $$\begin{aligned} m_{\tilde{g}}~\in & {} (1680, 2480) \; \mathrm{GeV}, \nonumber \\ m_{\tilde{q}}~\in & {} (1680, 2280) \; \mathrm{GeV}, \nonumber \\ m_{\tilde{\tau }_1}\in & {} (340, 500) \; \mathrm{GeV}, \nonumber \\ m_{\tilde{t}_1}\in & {} (810, 1110) \; \mathrm{GeV}, \end{aligned}$$

whereas the nominal values at the best-fit point are 2280, 2080, 450 and 1020 GeV, respectively. Since, in this optimistic scenario, squarks and gluinos would have been discovered previously at the LHC, measurements of their masses could be compared with the estimates based on the FCC-ee (TLEP) measurements. Agreement would constitute a non-trivial test of the CMSSM at the loop level, analogous to the tests of the Standard Model made possible by measurements of $m_t$ and $m_H$ and their consistency with predictions based on LEP and SLC data [61]. Conversely, any disagreement could be interpreted as a possible deviation from the CMSSM assumptions of universality for the soft supersymmetry-breaking parameters.

### Precision Higgs observables

We have made a similar estimate of the potential impact of the high-precision Higgs measurements possible with FCC-ee (TLEP) [65], as illustrated in the right panel of Fig. 14. In the upper left panel of Fig. 18 we display the deviation of the present experimental value of BR($H \rightarrow ZZ$) from the values calculated at points within the 68 and 95 % CL regions in the $(m_0, m_{1/2})$ plane of the CMSSM, in units of the present experimental error. In the other panels of Fig. 18 we show the numbers of FCC-ee (TLEP) $\sigma$’s by which the values of BR($H \rightarrow ZZ$) (upper right), BR($H \rightarrow WW$) (lower left) and BR($H \rightarrow gg$) (lower right) calculated at other points in the CMSSM $(m_0, m_{1/2})$ plane differ from the values at the low-mass CMSSM best-fit point.

As in the case of the electroweak precision measurements shown in Fig. 15, we see in the upper left panel of Fig. 18 that the entire 68 and 95 % CL regions of the CMSSM $(m_0, m_{1/2})$ plane lie within a single current LHC $\sigma$ of the present central value of BR($H \rightarrow ZZ$), and hence are shaded green, and we have checked that the same is true for the other Higgs branching ratios measured currently. For this reason, at the moment the Higgs branching ratios do not make important contributions to the global likelihood function of the CMSSM. However, we see in the other panels of Fig. 18 that future measurements of the Higgs branching ratios at FCC-ee (TLEP) would have the potential to discriminate between different CMSSM parameter sets, so that much of the 68 and 95 % CL regions in these panels are unshaded, since they lie more than three current standard deviations away from the prospective measurements. Specifically, several individual measurements at the central values predicted by the low-mass best-fit point in the CMSSM would each individually exclude regions at large values of $m_0$ and (particularly) $m_{1/2}$. As in Fig. 15 for the electroweak precision observables, we see that prospective measurements of the observables studied are compatible with the low-mass best-fit values within one FCC-ee (TLEP) $\sigma$ only within fractions of the ‘Crimean’ 68  % CL region. We also see that only narrow bands of the ‘Eurasian’ 95 % CL regions would yield values of BR($H \rightarrow ZZ, \gamma \gamma$) and BR($H \rightarrow gg$) within one $\sigma$ of the low-mass best-fit prediction, and that the band for BR($H \rightarrow gg$) does not overlap the others.

**Fig. 19:**
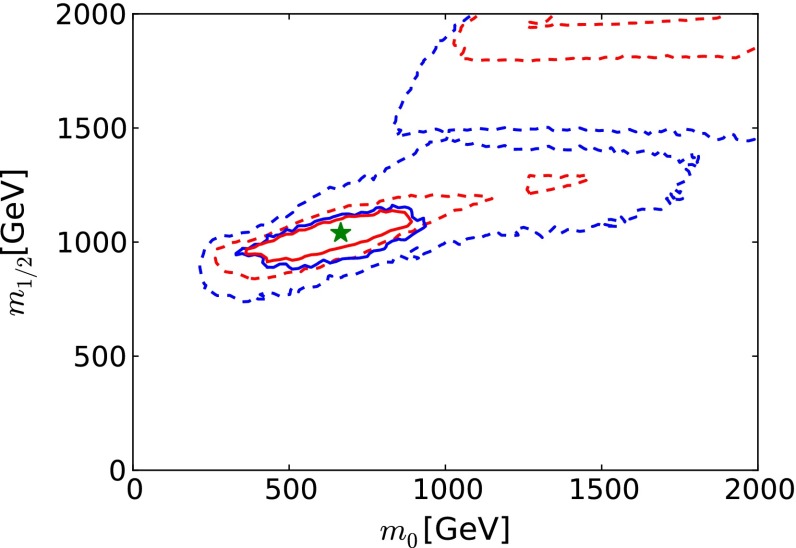
The $\Delta \chi ^2 = 2.30$ (68 % CL) and $\Delta \chi ^2 = 5.99$ (95 % CL) contours (*red* and *blue*, respectively) in the $(m_0, m_{1/2})$ plane for the CMSSM, assuming that Higgs measurements at FCC-ee (TLEP) [65] have the same central values as at the current low-mass best-fit points, and neglecting inevitable improvements in other constraints on the supersymmetric models

Also as in the case of the electroweak precision observables discussed above, we have made a crude estimate of the impact of the prospective FCC-ee (TLEP) Higgs measurements [65] on the global $\chi ^2$ function for the CMSSM, again neglecting the inevitable improvements in flavour and dark matter observables, and setting aside the electroweak precision observables as well as the direct measurements of sparticle masses. As we see in Fig. 19, the high-precision Higgs measurements would, by themselves, again provide constraints on $m_0$ and $m_{1/2}$, which would be of comparable importance to those from the electroweak precision observables shown in the corresponding panel of Fig. 16.

We display as solid blue lines in Fig. 17 the corresponding one-dimensional projections of the contribution of prospective FCC-ee (TLEP) Higgs measurements to the CMSSM global $\chi ^2$ function for $m_{\tilde{g}}$ (upper left panel), the generic squark mass $m_{\tilde{q}}$ (upper right panel), the lighter stop squark mass $m_{\tilde{t}_1}$ (lower left panel) and the lighter stau mass $m_{\tilde{\tau }_1}$ (lower right panel). We see that the Higgs estimates are comparable with the corresponding one-dimensional projections of the contribution of prospective FCC-ee (TLEP) electroweak precision measurements, shown as solid lines in the various panels of Fig. 17. The corresponding 95 % CL mass ranges are estimated to be

12 $$\begin{aligned} m_{\tilde{g}}~\in & {} (1980, 2500) \; \mathrm{GeV}, \nonumber \\ m_{\tilde{q}}~\in & {} (1780, 2320) \; \mathrm{GeV}, \nonumber \\ m_{\tilde{\tau }_1}\in & {} (370, 510) \; \mathrm{GeV}, \nonumber \\ m_{\tilde{t}_1}\in & {} (890, 1170) \; \mathrm{GeV}, \end{aligned}$$

whereas the nominal values at the best-fit point are 2280, 2080, 450 and 1020 GeV, respectively. The Higgs measurements clearly add another dimension to the tests of supersymmetric models at the loop level.

**Fig. 20:**
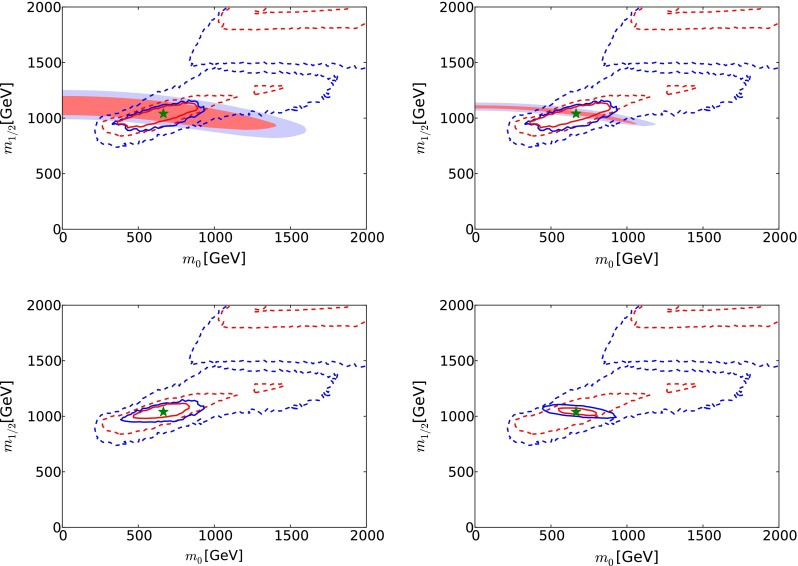
*Upper panels* the 68 and 95 % CL regions in the $(m_0, m_{1/2})$ plane of the CMSSM, overlaying potential direct measurements at LHC14 with 300/fb (*left panel*) and 3000/fb (*right panel*) (*pink* and *blue* shading) with indirect determinations via electroweak precision and Higgs measurements at FCC-ee (TLEP) [65] (*red* and *blue solid lines*). *Lower panels* the 68 and 95 % CL contours for the combination of the prospective constraints from LHC14 with 300/fb (*left panel*) and 3000/fb (*right panel*) with the indirect determinations via electroweak precision and Higgs measurements at FCC-ee (TLEP) [65] (*red* and *blue solid lines*). Also shown are the 68 and 95 % CL regions found in a recent global fit to the CMSSM (*red* and *blue dashed lines*) [33]

### Comparison between LHC and $e^+ e^-$ measurements

The potential comparison between LHC and FCC-ee (TLEP) measurements in the best-fit low-mass CMSSM scenario can be seen in Fig. 20, where we overlay in the CMSSM $(m_0, m_{1/2})$ plane the potential direct measurements at the LHC presented earlier (pink and blue shading) with indirect determinations at FCC-ee (TLEP) via EWPOs and Higgs measurements. A triple coincidence of direct sparticle mass measurements with indirect predictions from EWPOs and Higgs measurements would be truly impressive, a worthy successor to the successful predictions of the top and Higgs masses based on electroweak precision observables at LEP.

### Probes of grand unification

The precision measurements of electroweak precision observables and the strong coupling $\alpha _s(m_Z)$ at LEP and the SLC opened a new chapter in probes of models of grand unification [120–124], making possible for the first time a clear discrimination between the predictions of supersymmetric and non-supersymmetric scenarios. As remarked in [65], it is clear that FCC-ee (TLEP) measurements could take this confrontation between experiment and different grand unified theories to a completely new level, through more accurate determinations of the SU(3), SU(2) and U(1) couplings by specifying the supersymmetric spectrum and hence TeV-scale threshold corrections to the running of the couplings. The combination of these measurements would enable powerful constraints to be placed on the GUT-scale particles in any specific GUT model.

As an indication of this possibility, we consider the simplest supersymmetric SU(5) GUT, in which the GUT-scale particles comprise the heavy vector bosons *V*, the 24-plet Higgs bosons $\Sigma$ and the coloured Higgs triplet bosons $H_c$. By considering the three one-loop renormalisation-group equations (RGEs) for the SU(3), SU(2) and U(1) couplings in this model, Murayama and Pierce [125]^11^ derived the following one-loop relation between the low-energy values of the couplings, $\alpha _i(m_Z)$, the supersymmetric threshold and the mass of the Higgs triplet:

13 $$\begin{aligned}&- \frac{2}{\alpha _3(m_Z)} + \frac{3}{\alpha _2(m_Z)} - \frac{1}{\alpha _1(m_Z)} \nonumber \\&\quad = \frac{1}{2 \pi } \left[ \frac{12}{5} \ln \left( \frac{m_{H_c}}{m_Z}\right) - 2 \ln \left( \frac{m_{SUSY}}{m_Z} \right) \right] . \end{aligned}$$

Another combination of the one-loop RGEs gives a similar relation for a combination of the masses of the heavy vector bosons *V* and the 24-plet Higgs bosons $\Sigma$:

14 $$\begin{aligned}&\frac{2}{\alpha _3(m_Z)} + \frac{3}{\alpha _2(m_Z)} - \frac{5}{\alpha _1(m_Z)} \nonumber \\&\quad = \frac{1}{2 \pi } \left[ - 12 \ln \left( \frac{m^2_{V} m_\Sigma }{m^3_Z}\right) - 8 \ln \left( \frac{m_{SUSY}}{m_Z} \right) \right] . \end{aligned}$$

These relations are subject to corrections from higher-order terms in the RGEs, etc., but they may be used to estimate the uncertainties in the GUT-scale masses associated with uncertainties in the low-scale inputs.

For example, inverting (13) we find

15 $$\begin{aligned} \frac{\Delta m_{H_c}}{m_{H_c}} \;&\ni \; \frac{5}{6} \frac{\Delta m_{SUSY}}{m_{SUSY}} - \frac{5 \pi }{3} \Delta \left( \frac{1}{\alpha _3 (m_Z)} \right) \nonumber \\&\quad + \frac{5 \pi }{2} \Delta \left( \frac{1}{\alpha _2 (m_Z)} \right) - \frac{5 \pi }{6} \Delta \left( \frac{1}{\alpha _1 (m_Z)} \right) , \end{aligned}$$

and inverting (14) we find

16 $$\begin{aligned} 2 \frac{\Delta m_V}{m_V}&+ \frac{\Delta m_\Sigma }{m_\Sigma } \; \ni \; - \frac{2}{3} \frac{\Delta m_{SUSY}}{m_{SUSY}} - \frac{\pi }{2} \Delta \left( \frac{1}{\alpha _3 (m_Z)} \right) \nonumber \\&- \frac{\pi }{3} \Delta \left( \frac{1}{\alpha _2 (m_Z)} \right) + \frac{5 \pi }{6} \Delta \left( \frac{1}{\alpha _1(m_Z)} \right) . \end{aligned}$$

It is estimated that at FCC-ee one could attain uncertainties $\Delta \alpha _3 (m_Z) \sim 10^{-4}$ (corresponding to $\Delta \alpha _3^{-1} (m_Z) \sim 10^{-2}$) and $\Delta \sin ^2 \theta _W \simeq 10^{-6}$, with an input parametric uncertainty $\Delta \alpha _{em}^{-1} (m_Z) \simeq 5 \times 10^{-5}$. Using

17 $$\begin{aligned} \frac{1}{\alpha _2 (m_Z)} \; = \; \frac{\sin ^2 \theta _W}{\alpha _{em}(m_Z)}, \end{aligned}$$

we find that

18 $$\begin{aligned} \Delta \left( \frac{1}{\alpha _2 (m_Z)} \right)= & {} \Delta \sin ^2 \theta _W \times \frac{1}{\alpha _{em}(m_Z)} \nonumber \\&+ \sin ^2 \theta _W \times \Delta \left( \frac{1}{\alpha _{em} (m_Z)} \right) , \end{aligned}$$

and we infer that the dominant uncertainty in $\alpha _2^{-1} (m_Z)$ is that due to $\Delta \sin ^2 \theta _W$, giving us the estimate

19 $$\begin{aligned} \Delta \left( \frac{1}{\alpha _2 (m_Z)} \right) \; \sim \; 10^{-4}. \end{aligned}$$

Using then the relationship

20 $$\begin{aligned} \frac{1}{\alpha _1 (m_Z)} \; = \; \frac{ 3 \cot ^2 \theta _W}{5} \times \frac{1}{\alpha _2 (m_Z)}, \end{aligned}$$

it is evident that the dominant uncertainty in $\alpha _1^{-1} (m_Z)$ is correlated with that in $\alpha _2^{-1} (m_Z)$:

21 $$\begin{aligned} \Delta \left( \frac{1}{\alpha _1 (m_Z)} \right) \; \simeq \; \frac{ 3 \cot ^2 \theta _W}{5} \times \Delta \left( \frac{1}{\alpha _2 (m_Z)} \right) \; \simeq 2 \times 10^{-4}. \end{aligned}$$

It is apparent from the estimates (19) and (21) that the uncertainties due to the electroweak couplings in the GUT mass estimates (15) and (16) are much smaller than the uncertainties due to the strong coupling.

Specifically, the precision measurements at FCC-ee (TLEP) should enable the mass of the colour triplet to be estimated with an accuracy at the percent level, and similarly for the combination $m_V^2 m_\Sigma$, assuming that $m_{SUSY}$ can be determined with similar (or better) precision via direct or indirect measurements. Needless to say, this possibility of constraining GUT-scale masses would apply within a specific GUT model, and the implications of the FCC-ee (TLEP) measurements would depend on the model. However, this analysis makes the point that high-precision measurements with FCC-ee (TLEP) could impose important constraints on GUT models, taking to the next level the insights provided previously by LEP measurements [120–124].

## Prospects for the discovery of supersymmetry in pessimistic scenarios

We now consider the prospects for discovering supersymmetry in ‘pessimistic’ high-mass CMSSM scenarios in which the HL-LHC does not discover supersymmetry but provides only 95 % CL lower limits on the model parameters.

### Impact of LHC searches

**Fig. 21:**
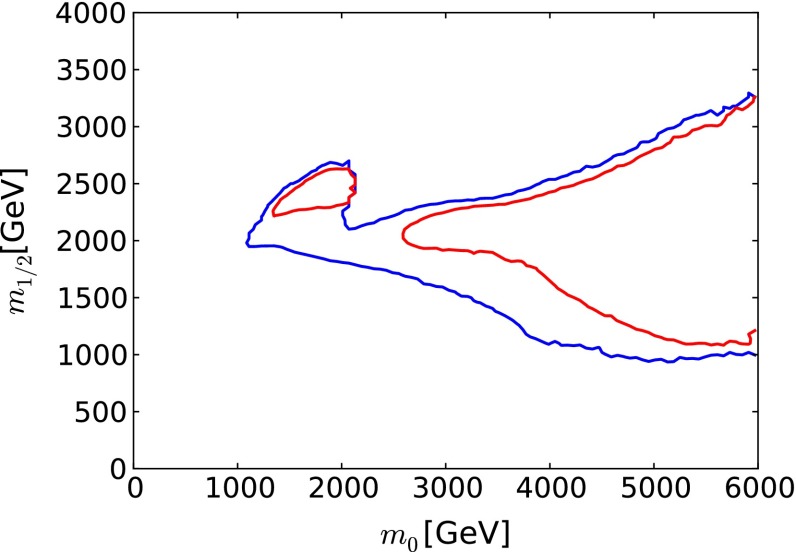
The $\Delta \chi ^2 = 2.30$ (68 % CL) and $\Delta \chi ^2 = 5.99$ (95 % CL) contours (*red* and *blue*, respectively) in the $(m_0, m_{1/2})$ plane for the CMSSM, assuming that supersymmetry has not been discovered at the LHC with 3000/fb of luminosity, and neglecting inevitable improvements in other constraints on the supersymmetric models

To probe this case, we first make a crude estimate of the impact of such a negative result by including in the global $\chi ^2$ functions for the CMSSM contributions based on the green lines in Fig. 1, which correspond to 95 % CL exclusion by the LHC with 3000/fb of data, neglecting again the inevitable improvements in the measurements of other observables that would provide additional constraints on supersymmetry. The resulting 68 and 95 % CL contours (red and blue, respectively) are shown in Fig. 21. The ‘Crimea’ region has now disappeared completely, and only the ‘Eurasia’ region remains. However, in the CMSSM, although this region is unified at the 95 % CL, it is divided at the 68 % CL into regions at lower and higher values of $m_0$.

**Fig. 22:**
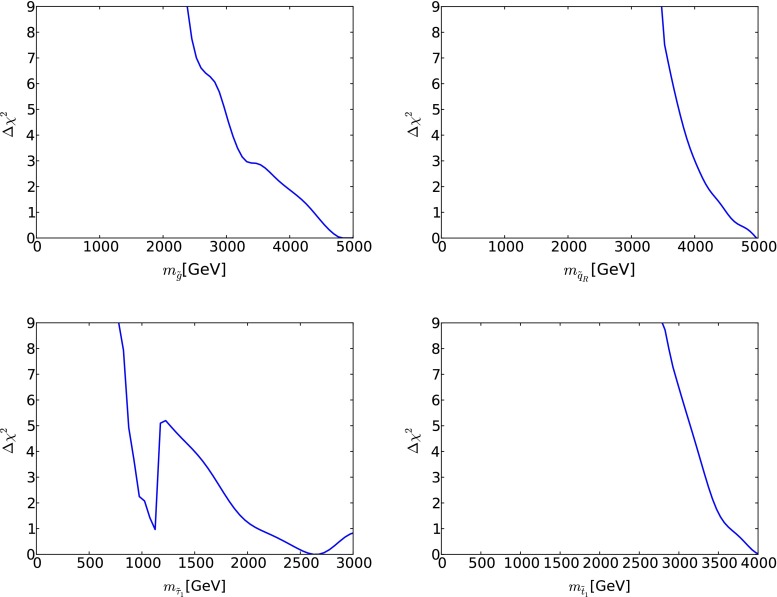
The one-dimensional profile likelihood functions for $m_{\tilde{g}}$ (*upper left panel*), $m_{\tilde{q}}$ (*upper right panel*), $m_{\tilde{\tau }_1}$ (*lower left panel*) and $m_{\tilde{t}_1}$ (*lower right panel*) in the CMSSM, assuming that supersymmetry has not been discovered at the LHC with 3000/fb of luminosity, and neglecting inevitable improvements in other constraints on the supersymmetric models

Figure 22 shows the corresponding one-dimensional profile likelihood functions for $m_{\tilde{g}}$ (upper left panel) and $m_{\tilde{q}}$ (upper right panel). In the case of the gluino, we find a prospective 95 % CL lower limit $m_{\tilde{g}} \gtrsim 3$ TeV, and a lower limit $m_{\tilde{q}} \gtrsim 4$ TeV for the squark mass. The limitations of our CMSSM sample [33] are such that we do not have any useful information as regards the likelihood functions for large masses, where they are expected to be quite flat. The lower left panel of Fig. 22 shows the corresponding one-dimensional profile likelihood function for $m_{\tilde{\tau }_1}$: the dip at $m_{\tilde{\tau }_1} \sim 1000$ GeV corresponds to the ‘cockscomb’ feature visible as an isolated 68 % CL region with $(m_0, m_{1/2}) \sim (1800, 2400)$ GeV in Fig. 21, with the local peak at $m_{\tilde{\tau }_1} \sim 1300$ GeV corresponding to the gap between the ‘cockscomb’ and the Eurasia region. As is also apparent in Fig. 13, within the CMSSM there is significant likelihood that $m_{\tilde{\tau }_1} < 1500$ GeV, so that ${\tilde{\tau }_1}$ pair production would be possible at CLIC, even if the LHC fails to discover supersymmetry.

### Direct sparticle searches at a higher-energy proton–proton collider

We now turn to the potential of a future higher-energy hadron collider for discovering supersymmetry within the CMSSM framework. To this end, we first analyse the nature of the CMSSM parameter space for large values of $m_0$ and $m_{1/2}$, taking into account the cold dark matter density constraint and the measurement of $m_h$, which are the only constraints capable of imposing upper limits on $m_0$ and $m_{1/2}$. Generally speaking, bringing the relic density down into the astrophysical range when these mass parameters are large requires some specific features in the sparticle spectrum such as near-degeneracy between the LSP, the NLSP and perhaps other supersymmetric particles, so as to suppress the relic LSP density via coannihilation, or the existence of a massive Higgs boson that acts as an s-channel resonance and thereby suppresses the LSP density by enhancing LSP annihilation.

One such possibility is the stau-coannihilation strip [127–133], which appears at low values of the ratio $m_0/m_{1/2}$, adjacent to the stau LSP region. Its length depends on $\tan \beta$ and $A_0$, extending as far as $m_{1/2} \sim 1400$ GeV for $\tan \beta = 40$ and $A_0 = 2.5 m_0$ [29]. A recent study has shown that all this strip may be explored by Run 2 of the LHC at 14 TeV [30], as also discussed above.

**Fig. 23:**
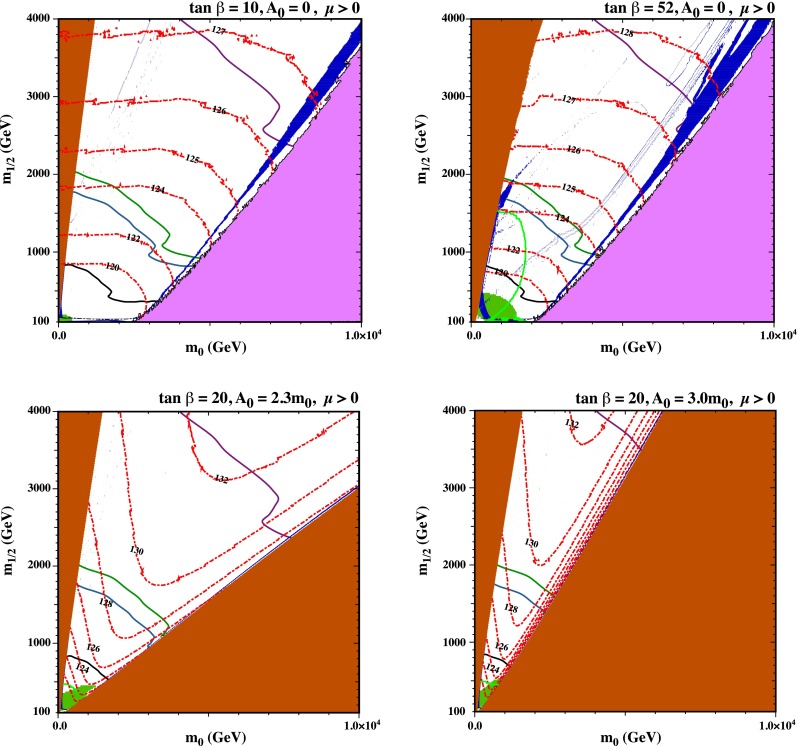
*Upper panels* the $(m_0, m_{1/2})$ planes for $A_0 = 0$ and $\tan \beta = 10$ (*left panel*) and 40 (*right panel*), displaying focus-point strips. *Lower panels* the $(m_0, m_{1/2})$ planes for $\tan \beta = 20$ and $A_0/m_0 = 2.3$ (*left panel*), and $A_0/m_0 = 3.0$ (*right panel*). In *each panel*, the ochre regions are excluded because of a charged LSP and/or a tachyon, and the *green regions* are excluded by $b \rightarrow s \gamma$ decay. There is no consistent electroweak vacuum in the *purple regions* in the *upper panels*. In the *dark blue strips* the relic LSP density lies within the range allowed by cosmology, and the *dashed red lines* are contours of $m_h$ as calculated using FeynHiggs 2.10.0. The *solid black*, *blue*, *green* and *purple lines* in *each panel* are particle exclusion reaches for $/ E_T$ searches with LHC at 8 TeV, 300 and 3000/fb with LHC at 14 TeV, and 3000/fb with HE-LHC at 33 TeV, respectively

Another possibility is the focus-point strip [56, 57, 134–137], which appears at higher values of the ratio $m_0/m_{1/2}$, adjacent to the boundary of the region where one can find a consistent electroweak vacuum, and generally lies beyond the reach of the LHC searches discussed earlier. Along this strip, the Higgsino component of the neutralino LSP is enhanced, and its annihilations and coannihilations with heavier neutralinos and charginos are enhanced. Various studies have shown that the focus-point strip may extend to very large values of $m_0$ and $m_{1/2}$, with $m_0/m_{1/2} \sim 3$ and $A_0 \lesssim m_0$. The upper panels of Fig. 23 display a pair of focus-point strips for $A_0 = 0$ and $\tan \beta = 10$ (left panel) and 52 (right panel), assuming $m_t = 173.2$ GeV.^12^ The regions of these planes where there is no consistent electroweak vacuum are coloured purple, the ochre regions at lower $m_0/m_{1/2}$ are excluded because of a charged LSP and/or a tachyon, and the green shaded regions are excluded by $b \rightarrow s \gamma$ decay.^13^

In both upper panels of Fig. 23, we see a (dark blue) focus-point strip hugging the boundary of the region at $m_0/m_{1/2} \gtrsim 3$ where electroweak symmetry-breaking is not possible. In the right panel, we also see a rapid-annihilation funnel [5–9] projecting out of the stau-coannihilation strip at low $m_0/m_{1/2}$ and extending to $(m_0, m_{1/2}) \sim (2500, 1800)$ GeV. We also see in both panels that the contours of $m_h$ calculated using FeynHiggs 2.10.0 [91] (shown as red dashed lines) are almost orthogonal to the focus-point strips. We note that the LHC measurement of $m_h$, even allowing for a 3 GeV uncertainty in the FeynHiggs 2.10.0 calculation, excludes $m_{1/2} \gtrsim 4000$ GeV. The solid black, blue, green and purple lines in each panel are particle exclusion reaches for $/ E_T$ searches with LHC at 8 TeV, 300 and 3000/fb with LHC at 14 TeV, and 3000/fb with HE-LHC at 33 TeV, respectively. The focus-point strip extends beyond the reach of the LHC, even with 3000/fb at 14 TeV in the centre of mass, and even beyond the reach of the HE-LHC with 3000/fb at 33 TeV. However, the portion allowed by the Higgs mass constraint lies comfortably within the reach of the FCC-hh with 3000/fb at 100 TeV, as discussed below.

As shown by two examples in the lower panels of Fig. 23, at larger values of $A_0/m_0 \gtrsim 2.2$ there are wedges at larger $m_0/m_{1/2}$, which are excluded because the lighter stop squark is the LSP. Hugging the boundaries of these wedges there are narrow stop-coannihilation strips [58, 138–143] where $\delta m_{\tilde{t}_1} - m_\chi$ is small. The opening angles of the stop LSP wedges have little dependence on $\tan \beta$, and both planes we show have $\tan \beta = 20$. On the other hand, the opening angles of the stop LSP wedges increase with $A_0/m_0$, with the result that the wedge at intermediate $m_0/m_{1/2}$ where the LSP is the lightest neutralino is closed off for $A_0/m_0 \gtrsim 5.5$.

**Fig. 24:**
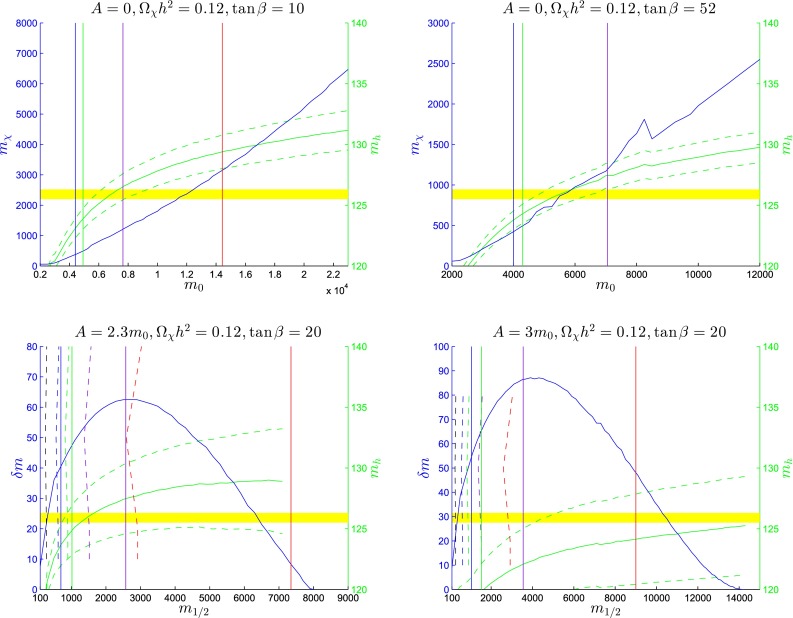
*Upper panels* the *solid blue lines* are the profiles in the $(m_0, m_\chi )$ plane of the focus-point strips for $A_0 = 0$ and $\tan \beta = 10$ (*left panel*), and $A_0 = 0$ and $\tan \beta = 52$ (*right panel*). *Lower panels* the *solid blue lines* are the profiles in the $(m_{1/2}, \delta m \equiv m_{\tilde{t}_1} - m_\chi )$ plane of the stop-coannihilation strips for $A_0/m_0 = 2.3$ and $\tan \beta = 20$ (*left panel*), and $A_0/m_0 = 3.0$ and $\tan \beta = 20$ (*right panel*). The near-vertical *black*, *blue*, *green*, *purple* and *red lines* in *each panel* are particle exclusion reaches for particle searches with LHC at 8 TeV, 300 and 3000/fb with LHC at 14 TeV, 3000/fb with HE-LHC at 33 TeV and 3000/fb with FCC-hh at 100 TeV, respectively. The *solid lines* are for generic $/ E_T$ searches, and (in the *lower panels*) the *dashed lines* are for dedicated stop searches. The *solid* (*dashed*) near-horizontal *green lines* are central values (probable ranges) of $m_h$ calculated using FeynHiggs 2.10.0, and the *yellow band* represents the experimental value of $m_h$

We display in the lower panels of Fig. 23 the cases $\tan \beta = 2.3$ (left panel) and 3 (right panel). As we discuss later, the stop-coannihilation strips extend far beyond the ranges of $m_0$ and $m_{1/2}$ that we display in these two panels of Fig. 23. These panels also display contours of $m_h$ calculated using FeynHiggs 2.10.0. We note that, in contrast to the focus-point cases displayed in Fig. 23, the $m_h$ contours cross the dark matter strip at a much smaller angle. As a consequence, the allowed ranges of $m_0$ and $m_{1/2}$ are much larger than in the focus-point case, after allowing for the 3 GeV uncertainty estimated within FeynHiggs 2.10.0 for a given input SLHA file [144, 145]. Additionally, we find that the value $m_h$ calculated along the stop-coannihilation strip is very sensitive to the codes used to evolve the CMSSM input parameters down to low energies and calculate the spectra used as SLHA inputs in the FeynHiggs 2.10.0 calculation of $m_h$. This introduces an additional uncertainty of several GeV in the value of $m_h$ corresponding to any given set of CMSSM input parameters. For this reason, no portions of these stop-coannihilation strips can be excluded on the basis of the LHC measurement of $m_h$.^14^

As in the upper panels of Fig. 23, the solid black, blue, green and purple lines in the lower panels are particle exclusion reaches for $/ E_T$ searches with LHC at 8 TeV, 300 and 3000/fb with LHC at 14 TeV, and 3000/fb with HE-LHC at 33 TeV, respectively, now for CMSSM scenarios with stop-coannihilation strips. We see that the LHC sensitivity contours in the lower panels of Fig. 23 include only portions of these stop coannihilation strips extending to $m_{1/2} \sim 500 \, (700)$ GeV for $\tan \beta = 20$ and $A_0 = 2.3 \, (3) \, m_0$. The HE-LHC sensitivity contour with 3000/fb at 33 TeV extends to $m_{1/2} \sim 2400 \, (3500)$ GeV along the stop-coannihilation strips for $\tan \beta = 20$ and $A_0 = 2.3 \, (3) \, m_0$, but large fractions of these strips lie beyond its reach.

**Fig. 25:**
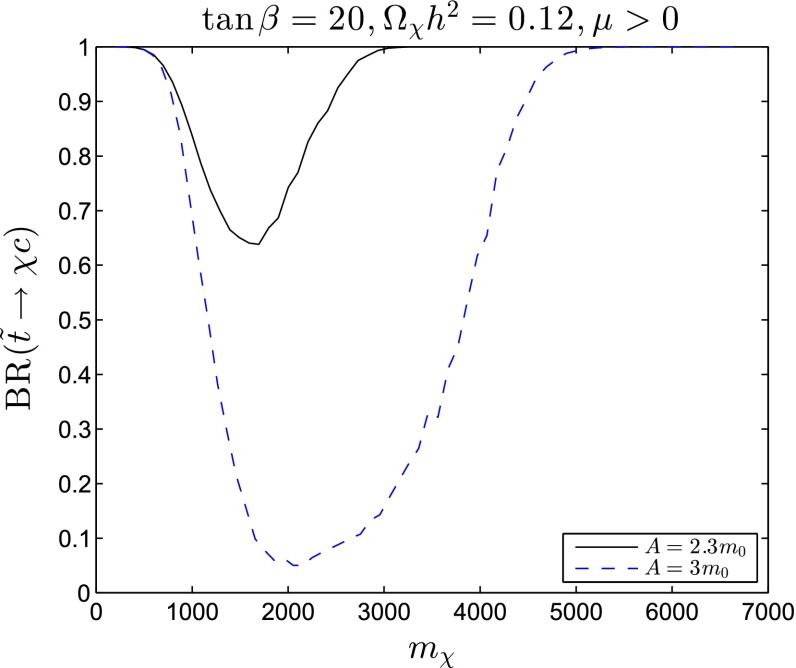
The branching ratio for ${\tilde{t}_1} \rightarrow \chi + c$ along the stop-coannihilation strips for $\tan \beta = 20$ and $A_0/m_0 = 2.3$ (*solid black line*) and $A_0/m_0 = 3.0$ (*dashed blue line*). In the latter case the branching ratio drops to a minimum $< 0.1$ when $m_{\tilde{t}_1} - m_\chi > m_W + m_b$, as seen in the lower right panel of Fig. 24

Figure 24 displays the profiles of the focus-point strips in Fig. 23 (upper panels) and of the stop-coannihilation strips in Fig. 23 (lower panels), along their full lengths. Both pairs of profiles exhibit the values of $m_h$ calculated using SLHA files obtained using SSARD as inputs to FeynHiggs 2.10.0 (near-horizontal solid green lines), including uncertainty estimates of $\pm 3$ GeV (near-horizontal dashed green lines). As already noted, only portions of the focus-point strips are compatible with the LHC measurement of $m_h$ (yellow bands) within these uncertainties, whereas in the cases of the stop-coannihilation strips there are significant additional uncertainties associated with the RGE running, and all portions of the strips are compatible with $m_h$. In the cases of the stop-coannihilation strips in the lower panels of Fig. 24, we also display as blue lines the mass difference $\delta m \equiv m_{\tilde{t}_1} - m_\chi$ along the strips.^15^ In the examples shown, this mass difference is generally $< m_W + m_b$, so that the branching ratio for two-body ${\tilde{t}_1} \rightarrow \chi + c$ decay usually dominates over that for four-body ${\tilde{t}_1} \rightarrow \chi + W + b + \nu$ decay. However, this is not always the case, as illustrated by examples in [58] and by Fig. 25 for the stop-coannihilation strip with $A_0/m_0 = 3.0$ and $\tan \beta = 20$. The branching ratio for ${\tilde{t}_1} \rightarrow \chi + W + b + \nu$ decay may dominate when $m_{\tilde{t}_1} - m_\chi > m_W + m_b$, as seen in the lower right panel of Fig. 24. Thus, a complete search for supersymmetry at FCC-hh should include searches for both the ${\tilde{t}_1} \rightarrow \chi + c$ and the ${\tilde{t}_1} \rightarrow \chi + W + b + \nu$ decay signatures.

The (near-)vertical lines in Fig. 24 mark our estimates of the sensitivities of the LHC (black - 8 TeV, blue - 300/fb at 14 TeV, green - 3000/fb at 14 TeV), 3000/fb at HE-LHC (purple) and 3000/fb at FCC-hh (red) along the stop-coannihilation strips. The solid lines represent the extrapolated reaches of the generic jets + $/ E_T$ searches, and the dashed lines in the lower panels represent the extrapolated reaches of dedicated searches for ${\tilde{t}_1} \rightarrow c + \chi$ decays, which lose some sensitivity as $\delta m$ increases because of the increase in the ${\tilde{t}_1} \rightarrow \chi + W + b + \nu$ decay branching ratio. We see that the FCC-hh would be sensitive to the full extents of the focus-point strips (upper panels) and of the stop-coannihilation strip for $A_0 = 2.3 \, m_0$ (lower left panel), but not all the stop-coannihilation strip for $A_0 = 3.0 \, m_0$ (lower right panel): this is true in general for $A_0/m_0 \gtrsim 2.5$.

### Impacts of electroweak and Higgs precision observables

We have also studied the possible impact of EWPOs and Higgs precision measurements along the focus-point and stop-coannihilation strips discussed in the previous subsection. We find that the contributions to the global $\chi ^2$ function of the present EWPOs and Higgs measurements do not vary strongly along the strips, so do not discuss them further. Instead, we focus on the potential impacts of FCC-ee measurements, choosing benchmark points on these strips. These benchmark points are chosen to have values of $m_H$, as calculated with FeynHiggs 2.10.0, that are highly compatible with the central experimental value $m_h \simeq 125$ GeV.^16^

**Fig. 26:**
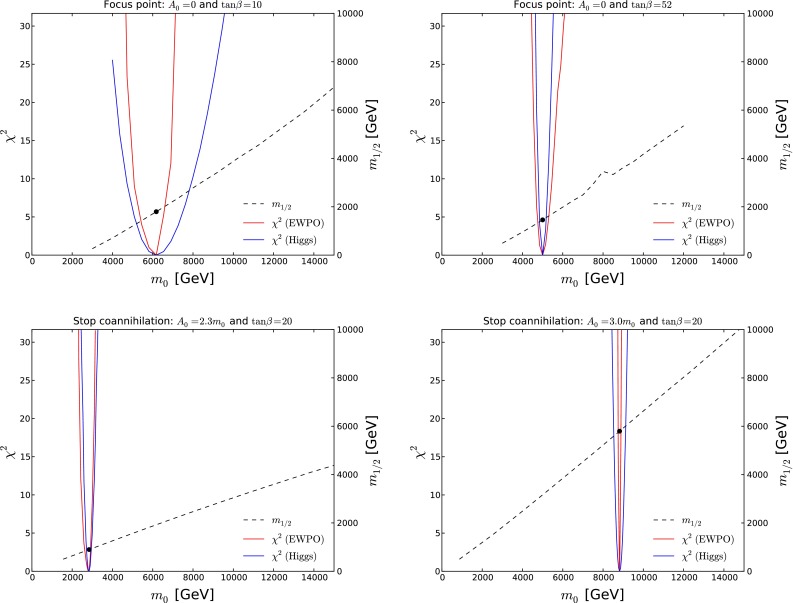
*Upper panels* the estimated contributions of the EWPOs and Higgs observables measured at FCC-ee (TLEP) to the global $\chi ^2$ function (*solid red* and *blue lines*, respectively) assuming the parameters of benchmark points along the focus-point strips for $A_0 = 0$ and $\tan \beta = 10$ (*left panel*), and $A_0 = 0$ and $\tan \beta = 52$ (*right panel*). *Lower panels* the same for the stop coannihilation strips for $A_0/m_0 = 2.3$ and $\tan \beta = 20$ (*left panel*), and $A_0/m_0 = 3.0$ and $\tan \beta = 20$ (*right panel*). The *diagonal black dashed lines* show the values of $m_{1/2}$ along the corresponding strips (*right-hand vertical axes*), and the black spots show the parameters of the corresponding benchmark points

Figure 26 shows the estimated contributions of the EWPOs and Higgs observables measured at FCC-ee (TLEP) (red and blue lines, respectively) to the global $\chi ^2$ functions along the focus-point and stop-coannihilation strips, which are plotted using $m_0$ as the horizontal axis. The diagonal dashed black lines show the corresponding values of $m_{1/2}$ using the scale shown on the right-hand vertical axis. In each case, we have assumed measurements with FCC-ee (TLEP) uncertainties and central values coinciding with those calculated using CMSSM model parameters at the benchmark points shown as the black spots. In each case, we see that the FCC-ee (TLEP) measurements would be capable of specifying with an accuracy that is greatest for the stop-coannihilation strip with $A_0/m_0 = 3.0$ and $\tan \beta = 20$ (lower right panel) and least for the focus-point strip with $A_0 = 0$ and $\tan \beta = 10$ (upper left panel).

All of these benchmark points lie within reach of generic $/ E_T$ searches at FCC-hh, as seen in Fig. 24. (Indeed, the fixed-point benchmark points lie with the reach of HE-LHC.) Therefore, for each of these benchmark points it would be possible to make a comparison between the direct determination of the CMSSM parameters with those inferred indirectly from FCC-ee measurements, much as we discussed earlier for the case of the low-mass CMSSM benchmark point, the LHC and FCC-ee. In all cases, FCC-ee would make possible tests of supersymmetry at the loop level, even in pessimistic scenarios where the LHC does not discover supersymmetry.

## Summary

We have explored in this paper the interplay between direct and indirect searches for supersymmetry in future runs of the LHC and at proposed future colliders. This is clearly a very broad topic, so we have restricted our attention to the CMSSM. A recent global fit to the CMSSM has found two favoured regions of its parameter space: a low-mass ‘Crimea’ region and a high-mass ‘Eurasia’ region. In the optimistic low-mass case, extrapolating the sensitivities of supersymmetry searches with LHC Run 1, we have found that future runs of the LHC with 300 or 3000/fb of data at 14 TeV should be able to discover gluinos and squarks if Nature is described by the CMSSM in the Crimea region. Moreover, the LHC experiments should be able to measure the gluino and squark masses with high accuracy and hence also the soft supersymmetry-breaking parameters of the CMSSM.

In this optimistic scenario, electroweakly interacting sparticles could be discovered at the CLIC $e^+ e^-$ collider and their masses measured very accurately, providing an important cross-check on the consistency of the CMSSM with its universal input soft supersymmetry-breaking parameters. On the other hand, an $e^+ e^-$ collider with centre-of-mass energy limited to 0.5 TeV would not produce supersymmetric particles even in this optimistic scenario, though a collider with a centre-of-mas energy of 1 TeV could detect the lighter stau slepton.

However, an $e^+ e^-$ collider capable of high-luminosity running on the *Z* peak as well as producing large numbers of Higgs bosons, such as FCC-ee (TLEP), would provide two sets of high-precision measurements that could be used to constrain supersymmetric loop corrections and hence, in conjunction with the LHC measurements, check supersymmetric predictions at the quantum level in two independent ways. The combination of direct and indirect measurements possible in this optimistic scenario would test the CMSSM in a way reminiscent of the use of precision measurements from LEP and elsewhere to predict successfully the masses of the top quark and the Higgs boson, albeit in time-reversed order.

In the pessimistic scenario in which sparticles are too heavy to be produced at the LHC, the CLIC $e^+ e^-$ collider might still be able to discover the lighter stau slepton. We have also assessed the capability of a higher-energy proton–proton collider such as FCC-hh to discover supersymmetry, and the ability of high-precision FCC-ee (TLEP) measurements to provide any indirect evidence. For this part of our analysis, we concentrate on the narrow strips in the CMSSM parameter space that extend to large sparticle masses, namely the stop-coannihilation strip and the focus-point strip. We find that a 33-TeV collider such as HE-LHC would be able to discover supersymmetry via $/ E_T$ searches along some fractions of these strips, and that a 100-TeV collider such as FCC-hh would be able to discover supersymmetry along most of the extents of the strips examined. By studying specific benchmark points along these strips, we have shown that FCC-ee measurements could in principle determine indirectly CMSSM model parameters also in the pessimistic scenario, and the combination with FCC-hh measurements could test supersymmetry at the loop level.

In both the optimistic and the pessimistic scenarios, we find that high-precision measurements with a high-luminosity $e^+ e^-$ collider can play rôles that are complementary to direct particle searches with proton–proton colliders, and they could enable supersymmetry to be tested at the quantum (loop) level). Run 2 of the LHC will provide us with some valuable pointers indicating which of these scenarios may be realised in Nature.

## Footnotes

1 This paper also contains a global fit to the NUHM1 [77–80], and a fit to the NUHM2 [79–82] can be found in [83], together with minor updates of these CMSSM and NUHM1 fits. A further update of the CMSSM analysis can be found in [84]. These updates do not impact qualitatively the analyses presented in this paper.

2 This discrepancy can be resolved in a model that relaxes the assumption of GUT-scale universality in the soft supersymmetry-breaking parameters [84].

3 We have verified that very similar results can be obtained using the NNPDF2.3LO, option 14 parton distribution functions [115].

4 There are also extrapolations available for slepton and chargino searches. However, in those cases the interpretations of the searches are more delicate [84], and we do not discuss them here.

5 We note that the relative fractions of the gluon–gluon, gluon–squark and squark–squark final states vary continuously in these plots.

6 We consider this to be a conservative assumption, as the signal-to-background ratio is likely to improve.

7 Here and subsequently, we include in our simulations the Standard Model backgrounds from $W^\pm$, $Z^0$, ${\bar{t}} t$ and single-*t* production. However, we do not embark on a simulation of either ATLAS or CMS, since the purpose of our exploratory study is to give a first feeling for what might be possible in future LHC runs, and suitable detector simulations for the high-luminosity LHC are not available.

8 The main effect of the measurement of the supplementary jet in ${\tilde{g}} \rightarrow {\tilde{q}_R} + q$ decay is to truncate the preferred strip in the 300/fb case: it has no visible effect in the 3000/fb case.

9 On the other hand, this would be possible within the pMSSM10 analysis discussed in [84].

10 As discussed later, the solid blue lines are the corresponding likelihood functions provided by the prospective Higgs measurements at FCC-ee (TLEP).

11 For a recent analysis, see [126].

12 Larger (smaller) values of $m_0/m_{1/2}$ are found for larger (smaller) values of $m_t$.

13 The lighter green line in the upper right panel of Fig. 23 delineates the region allowed by $B_s \rightarrow \mu ^+ \mu ^-$.

14 We assume in calculating these parameter planes that $\alpha _s (m_Z) = 0.1184$ and $\sin ^2 \theta _W = 0.2325$. With these choices, the SU(3), SU(2) and U(1) couplings are in general not quite equal at the GUT scale, but the deviations are generally compatible with zero when the uncertainties in the inputs are taken into account, and any apparent non-universality could be cancelled by GUT threshold corrections and/or produced by higher-dimensional operator contributions. The comparable planes shown in [58] assumed $\sin ^2 \theta _W = 0.2325$ and strict coupling-constant unification, and consequently had a range of values of $\alpha _s(m_Z)$. This is the reason for the differences between these planes and comparable planes in [58].

15 This is generally larger than in the cases with strict gauge coupling unification assumed in [58], with the corollary that the strips extend to larger $m_{1/2}$ than shown there.

16 We recall that calculations of $m_h$ with FeynHiggs 2.10.0 have uncertainties $\sim \pm 3$ GeV so that, as discussed in [58], substantial fractions of the focus-point and stop-coannihilation strips are compatible with the $m_h$ constraint.
